# Interaction between poly(A)–binding protein PABPC4 and nuclear receptor corepressor NCoR1 modulates a metabolic stress response

**DOI:** 10.1016/j.jbc.2023.104702

**Published:** 2023-04-12

**Authors:** A.G. Oliveira, L.D. Oliveira, M.V. Cruz, D.S.P.S.F. Guimarães, T.I. Lima, B.C. Santos-Fávero, A.D. Luchessi, B.A. Pauletti, A.P. Leme, M.C. Bajgelman, J. Afonso, L.C.A. Regitano, H.F. Carvalho, E.M. Carneiro, J. Kobarg, V. Perissi, J. Auwerx, L.R. Silveira

**Affiliations:** 1 Obesity and Comorbidities Research Center (OCRC), Department of Structural and Functional Biology, Institute of Biology (IB), University of Campinas (UNICAMP), Campinas, São Paulo, Brazil; 2 Department of Biochemistry, Boston University School of Medicine, Boston, Massachusetts, USA; 3 Laboratory of Biotechnology, School of Applied Sciences, University of Campinas (UNICAMP), Limeira, São Paulo, Brazil; 4 Brazilian National Laboratory for Biosciences (LNBio), Center for Research in Energy and Materials (CNPEM), Campinas, São Paulo, Brazil; 5 Empresa Brasileira de Pesquisa Agropecuária, Embrapa Pecuária Sudeste, São Carlos, Brazil; 6 Laboratory of Extracellular Matrix and Gene Regulation, Department of Structural and Functional Biology, Institute of Biology (IB), University of Campinas (UNICAMP), Campinas, São Paulo, Brazil; 7 Faculty of Pharmaceutic Sciences, University of Campinas (UNICAMP), Campinas, São Paulo, Brazil; 8 Laboratory of Integrative and Systems Physiology, École Polytechnique Fédérale de Lausanne, Lausanne, Switzerland

**Keywords:** metabolism, mitochondria, NCoR1, nuclear receptors, PABPC4, transcription corepressor

## Abstract

Mitochondria are organelles known primarily for generating ATP *via* the oxidative phosphorylation process. Environmental signals are sensed by whole organisms or cells and markedly affect this process, leading to alterations in gene transcription and, consequently, changes in mitochondrial function and biogenesis. The expression of mitochondrial genes is finely regulated by nuclear transcription factors, including nuclear receptors and their coregulators. Among the best-known coregulators is the nuclear receptor corepressor 1 (NCoR1). Muscle-specific knockout of NCoR1 in mice induces an oxidative phenotype, improving glucose and fatty acid metabolism. However, the mechanism by which NCoR1 is regulated remains elusive. In this work, we identified the poly(A)–binding protein 4 (PABPC4) as a new NCoR1 interactor. Unexpectedly, we found that silencing of PABPC4 induced an oxidative phenotype in both C2C12 and MEF cells, as indicated by increased oxygen consumption, mitochondria content, and reduced lactate production. Mechanistically, we demonstrated that PABPC4 silencing increased the ubiquitination and consequent degradation of NCoR1, leading to the derepression of PPAR-regulated genes. As a consequence, cells with PABPC4 silencing had a greater capacity to metabolize lipids, reduced intracellular lipid droplets, and reduced cell death. Interestingly, in conditions known to induce mitochondrial function and biogenesis, both mRNA expression and PABPC4 protein content were markedly reduced. Our study, therefore, suggests that the lowering of PABPC4 expression may represent an adaptive event required to induce mitochondrial activity in response to metabolic stress in skeletal muscle cells. As such, the NCoR1–PABPC4 interface might be a new road to the treatment of metabolic diseases.

Mitochondria are organelles known primarily for generating ATP through electron transfer coupled to the oxidative phosphorylation process. Environmental signals such as cold temperature and high energy demand affect this process in mammals leading to an increase in mitochondrial function and biogenesis (1). Precise coordination between the environmental signals and transcriptional regulation of gene expression is essential for this adaptive process (2), as it allows the cells to promptly regulate the expression of the gene sets regulating mitochondrial homeostasis (3). Conversely, dysfunction in this system has been associated with metabolic diseases such as insulin resistance and type 2 diabetes (4).

Mitochondrial proteins are encoded in both nuclear and mitochondrial genomes by a synchronized action of transcriptional factors and regulatory proteins that work in concert to adapt the cells to stressful conditions (3). Among others, the nuclear receptor corepressor 1 (NCoR1) is known to interact with transcription factors, including thyroid hormone receptor (TR), retinoic acid receptor, peroxisome proliferator-activated receptor (PPAR), and estrogen-related receptor, that regulate mitochondrial biogenesis and respiration through the expression of key nuclear-encoded mitochondrial genes (3, 5, 6). Accordingly, systemic knockout of NCoR1 in mice results in increased energy metabolism and oxygen consumption, an effect reflected in improved insulin sensitivity (2). Similarly, skeletal muscle–specific knockout of NCoR1 increased oxidative metabolism and improved insulin sensitivity in high-fat diet fed mice (7); and genetic ablation of NCoR1 in white adipose tissue decreases the inflammatory process, leading to improvement of insulin sensitivity (7). In contrast, overexpression of NCoR1 in C2C12 cells was associated with lower mitochondrial respiration, oxidative stress, and cell death, clearly demonstrating the role of NCoR1 in the regulation of metabolic and cellular homeostasis (8). However, the molecular regulation of NCoR1 remains unclear.

To address this question, we performed NCoR1 immunoprecipitation NCoR1-FLAG coupled with mass-spectrometry to identify new interactors with the potential to regulate its repressor activity. In addition to the classic NCoR1 interactors, poly(A)–binding protein cytoplasmic 4 (PABPC4) protein was identified. Humans encode at least five PABP isoforms that act in different compartments regulating multiple intracellular pathways (9). The most characterized is PABPC1, first demonstrated to promote the initiation of translation and RNA stability during termination of translation process and consequently preventing nonsense-mediated mRNA decay (10). The least studied isoform, PABPC4, is structurally and functionally similar to PABPC1 and is enriched in skeletal muscle (11). Unexpectedly, we observed that the expression of PABPC4 in C2C12 cells was reduced in response to a number of metabolic stresses, including low glucose, galactose, and mitochondrial chemical uncoupling. This effect was also observed in mice after a bout of acute exercise, suggesting that a reduced expression of PABPC4 contributes for mitochondrial adaptation in response to metabolic insults. Under such conditions, PPARβ activity is upregulated by a mechanism dependent on NCoR1 ubiquitination. Consistent with these findings, silencing of PABPC4 markedly induced mitochondrial activity, and this effect was associated with reduced intracellular lipid droplets and cell death. We, therefore, postulated that downregulation of PABPC4 may represent an adaptive event that contributes to the maintenance of mitochondrial homeostasis in skeletal muscle cells by promoting NCoR1 degradation.

## Results

### PABPC4 interact with NCoR1, and its abundance is reduced in response to metabolic stress

**Figure 1:**
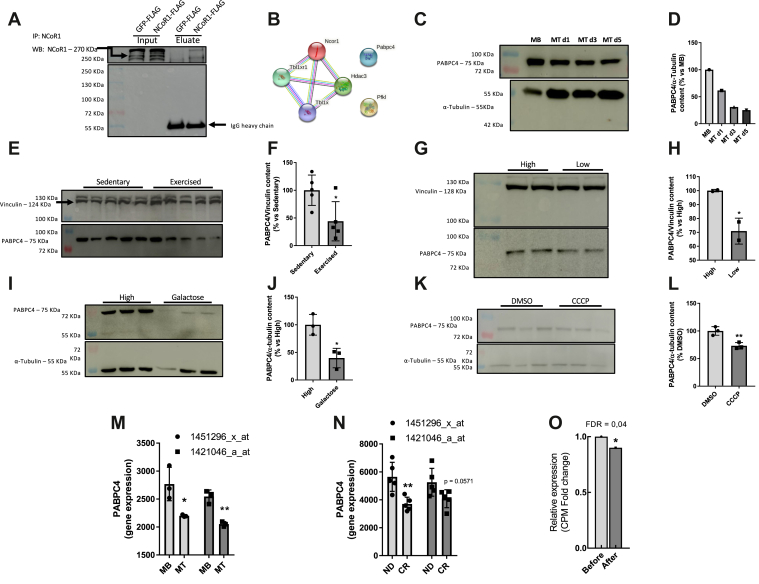
**PABPC4 interacts physically with NCoR1, and its genic expression and protein content are downregulated in response to metabolic stress.***A*, MEF cells were transiently transfected with pcDNA-GFP-FLAG (control) or pCMX-NCoR1-FLAG, and 48 h after transfection, the cells were lysed and samples subjected to mass spectrometry to the identification of NCoR1-interacting proteins. *B*, the interaction diagram depicts the already known and the NCoR1 interactors identified in this work. *C*, PABPC4 protein content was analyzed by Western blotting during C2C12 cell differentiation (n = 2 independent experiments and image representative of one experiment). MT d1, d3, and d5 = cells were collected 1, 3, or 5 days after the addition of the differentiation medium. *D*, bar graph representing the quantitation from (*C*). *E*, PABPC4 protein content analyzed by Western blotting in the red portion of the gastrocnemius muscle of sedentary and acutely exercised mice (n = 5). Mice run at 70% maximum VO_2_ until exhaustion. *F*, bar graph representing the quantitation from (*E*). *G*, PABPC4 protein content analyzed by Western blotting in C2C12 myotubes exposed to low glucose (5 mM) for 12 h (n = 2). *H*, bar graph representing the quantitation from (*G*). *I*, PABPC4 protein content analyzed by Western blotting in C2C12 myotubes treated with 10 mM galactose for 12 h (n = 3). *J*, bar graph representing the quantitation from (*I*). *K*, PABPC4 protein content was analyzed by Western blotting in C2C12 myotubes treated with 10 μM CCCP for 16 h (n = 3). *L*, bar graph representing the quantitation from (*K*). *M*, gene expression accessed by microarray retrieved from a publicly available dataset (accession number GDS2412), showing two different probes to PABPC4 during the C2C12 myogenesis process (n = 3). *N*, gene expression accessed by microarray retrieved from a publicly available dataset (accession number GSE6323) showing two different probes to PABPC4 in mice under normal diet (ND) or caloric restriction (CR) (n = 5). *O*, PABPC4 mRNA expression before and after exercise training in humans was performed by RNA-seq and retrieved from a publicly available dataset (accession number GSE60591). Bars represent the mean ± SD. ∗*p* < 0.05 and ∗∗*p* < 0.01 *versus* control. co-IP/mass spectrometry experiment was performed one time with three independent samples. CCCP, carbonyl cyanide 3-chlorophenylhydrazone; co-IP, coimmunoprecipitation; MEF, mouse embryonic fibroblastic; MB, myoblast; NCoR1, nuclear receptor corepressor 1; PABPC4, poly(A)–binding protein cytoplasmic 4.

Despite the fact that NCoR1 is a well-known corepressor in both adipose and muscle tissue, its regulation in muscle tissue is not fully understood (12, 13). To address this question, we first evaluated the effect of NCoR1 overexpression in mouse embryonic fibroblastic (MEF) cells. As expected, NCoR1 overexpression decreased the expression of PPAR gene targets (Fig. S1, *A* and *B*). Conversely, its knockdown (KD) increased the oxygen consumption rate (OCR, Fig. S1, *C* and *D*). This confirmed the functional importance of NCoR1 in regulating cellular and metabolic homeostasis in MEF cells. Despite a well-developed mitochondria network, MEF cells do not show the same metabolic flexibility as muscle cells, such as C2C12. In order to better understand how NCoR1 is regulated, we performed a coimmunoprecipitation-mass spectrometry (co-IP/mass spec) experiment using NCoR1 as bait to identify new interactors. The approach was validated by the recovery of known members of the NCoR1 complex, such as the histone deacetylase HDAC3 and the exchange factors TBL1X and TBLR1 (14). Besides these known interactors, we identified two new interactors, PABPC4 and PFKL (Fig. 1, *A* and *B*). The interaction between endogenous NCoR1 and PABPC4 was confirmed by a reverse co-IP, where PABPC4 was immunoprecipitated, and samples were probed with NCoR1 antibody (Fig. S1*E*). Given that PABPC4 interacts with mRNA, we performed another co-IP followed by RNAse treatment to rule out the possibility that PABPC4–NCoR1 interaction was mediated by mRNA. Indeed, we observed that the interaction between PABPC4 and NCoR1 is not mediated by RNA (Fig. S1*F*). Next, we searched for already-known NCoR1 and PABPC4 interactors using the human database in Biogrid (https://thebiogrid.org/). It allowed us to identify 18 common interactors for both PABPC4 and NCoR1 (Fig. S1, *G* and *H*), and most of these proteins are associated with response to estradiol or RNA metabolism (Fig. S1*I*). Once the interaction was confirmed, we analyzed PABPC4 under different metabolic stresses known to increase mitochondrial biogenesis or increase the oxidative phosphorylation capacity. These experiments revealed that PABPC4 was reduced in response to different stimuli associated with increased mitochondrial oxidative capacity (*e.g.*, cell differentiation, exercise, energetic stress, or mitochondrial uncoupling) (Fig. 1, *C*–*O*). These conditions were also associated with increased abundance of mitochondrial electron transport chain (ETC) proteins (Fig. S1, *J*–*O*).

### Characterization of NCoR1–PABPC4 interaction

**Figure 2:**
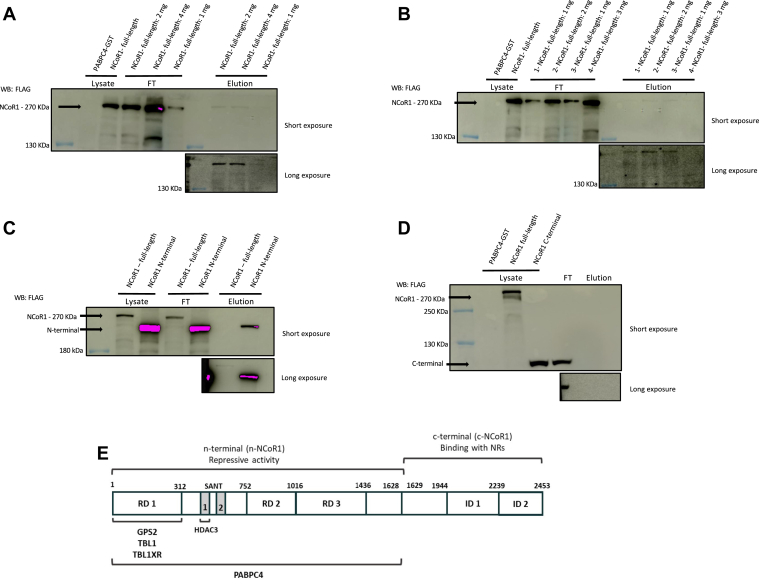
**Pull-down of PABPC4 and different NCoR1 constructions.** Hek293T cells were transiently transfected with pCMX-NCoR1-Flag (NCoR1 full-length), pCMV6–NCoR1 N-terminal-FLAG (NCoR1 N-terminal), or pCMV6–NCoR1 C-terminal-FLAG (NCoR1 C-terminal) plasmids. PABPC4-GST expression was performed in *Escherichia coli* and the lysate used in the pull-down assays. The glutathione agarose resin was functionalized by first binding 0.668 mg of PABPC4-GST. *A*, either bacteria expressing PABBPC4-GST or Hek293T cells expressing NCoR1 full-length were lysates. After that, different amounts of NCoR1 full-length were loaded into the column. Bacterial PABPC4-GST lysate was used to demonstrate that NCoR1 or any other bacterial protein interacts with PABPC4 and could be detected by the FLAG antibody. Different amounts of NCoR1 full-length (1, 2, or 4 mg) were loaded in the column containing PABPC4-GST and incubated as described in the Experimental procedures section. After that, the column was washed, and proteins were eluted and assayed by Western blotting. NCoR1 band was identified using an anti-FLAG antibody. *B*, the same experiment described in (*A*) but with different mass ratios between PABPC4-GST and NCoR1 full-length; 1: 0.668 mg of PABPC4-GST and 1 mg of NCoR1 full-length cell lysate; 2: 0.668 mg of PABPC4-GST and 2 mg NCoR1 full-length cell lysate; 3: 1 mg of PABPC4-GST and 1 mg of NCoR1 full-length cell lysate; 4: 1 mg of PABPC4-GST to 3 mg of NCoR1 full-length cell lysate. *C*, Hek293T cell expressing either NCoR1 full-length or NCoR1 C-terminal constructs were lysate, loaded into the column, washed, eluted, and then identified by Western blotting. NCoR1 band was identified using an anti-FLAG antibody. *D*, either bacteria expressing PABBPC4-GST or Hek293T cells expressing NCoR1 full-length or NCoR1 C-terminal were lysate. The lysates form both NCoR1 full-length and NCoR1 C-terminal were used in a pull-down assay. The NCoR1 band was identified using an anti-FLAG antibody. *E*, schematic representation depicting NCoR1 structure in mice and its binding partners. At the N-terminus (1-1622), it is shown the corepressor domains (RD1, RD2, and RD3) and also some of the proteins known to make up the core NCoR1 complex, such as HADH3, GPS2, TBL1 e TBL1XR1. At the C-terminus (1629-2453), it is shown the nuclear receptor–interaction domains (ID1 and ID2). Our results show that PABPC4 interacts with NCoR1 at its N-terminus region. FT, Flow-through (fraction not bound to column); NCoR1, nuclear receptor corepressor 1; PABPC4, poly(A)–binding protein cytoplasmic 4; WB, western blotting.

To further characterize the interaction between PABPC4 and NCoR1, we genetically manipulated Hek293T cells through the recombinant expression of either the full-length NCoR1 protein (1–2453aa), or the N-terminal (1–1628aa), or C-terminal portion (1629–2453aa). The pull-down experiments confirmed the interaction between PABPC4 and the entire NCoR1 protein and showed that the N-terminal portion is the NCoR1-interacting portion of PABPC4 (Fig. 2, *A*–*D*). To confirm that the interaction occurs directly between the two candidate proteins and not through tags or resin, control tests were performed, as shown in Fig. S2, *A*–*G*. Neither NCoR1 full length nor its portions interact with the GST-tag or pure resin. The FLAG-tag contained in NCoR1 and its portions were also evaluated through other tagged proteins, without any nonspecific interaction between the proteins and PABPC4, GST, or pure resin being observed.

### PABPC4 KD induces mitochondrial function and biogenesis

**Figure 3:**
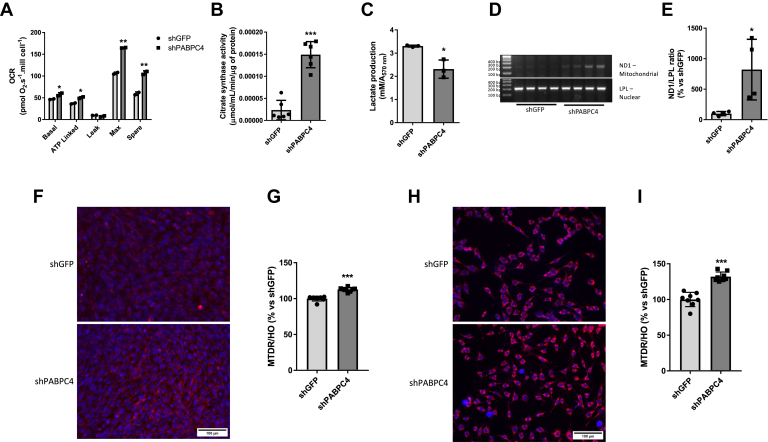
**PABPC4 knockdown increases the oxidative phenotype in both C2C12 and MEF cells.***A*, oxygen consumption rate (OCR) representative image (n = 2), (*B*) citrate synthase activity (n = 3), (*C*) lactate production (N = 3), (*D*) RT-PCR for nuclear (LPL) and mitochondrial-encoded gene (ND1) (n = 4), (*E*) bar graph representing the quantitation from (*D* and *F*) representative image using the mitochondrial probe MitoTracker Deep Red. Mitochondria were stained in *red*, and nuclei were stained with Hoechst 33342 (*blue*). Magnification = 200 ×. *G*, bar graph from (*F*) analysis performed in shGFP and shPABPC4 myotubes (n = 8). The fluorescence emitted by MitoTracker Deep Red (MTDR) was normalized by the fluorescence emitted by Hoechst 33342 (HO), and the results were expressed as a percentage related to shGFP. *H*, mitochondrial content staining using MitoTracker Deep Red in MEF shGFP and shPABPC4 cells (n = 8). Mitochondria were stained in *red*, and nuclei were stained with Hoechst 33342 (*blue*). Magnification = 200 ×. *I*, bar graph from (*H*) and expressed as described in (*G*). All experiments were repeated at least three times, except the mitochondrial DNA copy number, which was repeated twice. Bars represent the mean ± SD. ∗*p* < 0.05 *versus* shGFP; ∗∗*p* < 0.01 *versus* shGFP; ∗∗∗*p* < 0.001 *versus* shGFP. MEF, mouse embryonic fibroblastic; PABPC4, poly(A)–binding protein cytoplasmic 4.

We next aimed at understanding how this interaction affects the metabolic response and transcriptional process of genes regulated by NCoR1 in skeletal muscle cells. As the PABPC4 protein content was reduced in response to different metabolic stimuli associated with increased mitochondrial function and biogenesis, we asked whether the KD PABPC4 gene in both muscular and MEF cells would affect mitochondrial homeostasis (Fig. S3, *A*–*F*). As expected, PABPC4 KD increased basal, ATP-linked, maximal, and spare capacity OCR in myotubes (Fig. 3*A*) and MEF cells (Fig. S3*G*). Additionally, PABPC4 KD cells showed increased citrate synthase activity (Fig. 3*B*), decreased lactate production in both myotubes and MEF cells (Figs. 3*C* and S3*H*), and increased mitochondrial DNA copy number (Fig. 3, *D* and *E*). Mitochondrial content was also markedly increased as determined by a mitochondrial fluorescent dye in both myotubes (Fig. 3, *F* and *G*) and MEF cells (Fig. 3, *H* and *I*).

### PABPC1 is not associated with metabolic stress

**Figure 4:**
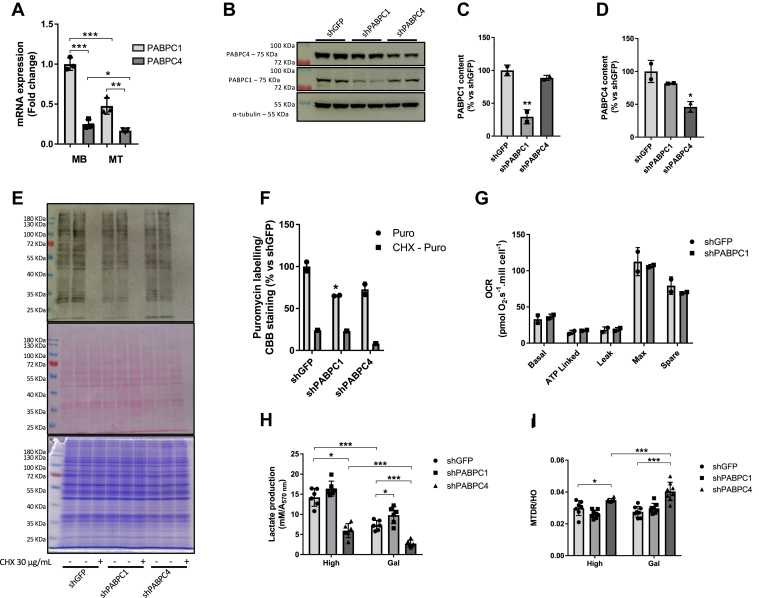
**PABPC1 do not play a role in mitochondria function in myotubes.***A*, PABPC1 and PABPC4 mRNA levels were assessed by RT-qPCR in both myoblast (MB) and myotubes (MT) (n = 3). Expression was calculated as fold change related to PABPC1 mRNA. *B*, C2C12 cells knockdown for either PABPC4 or PABPC1 were differentiated, lysed, and samples were subjected to Western blotting (n = 2). *C* and *D*, bar graph representing the PABPC1 or PABPC4, respectively, quantitation from (*B*). *E*, C2C12 cells knockdown for either PABPC4 or PABPC1 were seeded in 6-well plates and treated with 10 μg/ml of puromycin for 30 min before the analysis of protein synthesis. Prior addition of puromycin, 30 μg/ml cycloheximide (CHX) was added, for 30 min, to inhibit the protein synthesis process. *Upper panel*: Western blotting using anti-puromycin antibody; middle panel: Ponceau red–stained membrane from the same membrane probed with anti-puromycin antibody in the upper panel; lower panel: a Coomassie Brilliant Blue (CBB)-stained gel run in parallel and with the same samples as in upper panel (n = 2 for puromycin-treated cells; n = 1 for CHX pretreated cells). *F*, quantitation from (*E*). Quantitation was performed by normalizing the optical density from puromycin-labeled membrane divided by the optical density from the ponceau-stained membrane. *G*, oxygen consumption rate (OCR) in PABPC1 knockdown cells (n = 2). *H*, lactate production in PABPC1 or PABPC4 knockdown cells in high glucose (25 mM) or galactose (10 mM, no glucose), for 12 h (n = 4). *I*, mitochondrial content was analyzed using MitoTracker Deep Red (MTDR) staining in cells exposed to high glucose (25 mM) or galactose (10 mM, no glucose), for 12 h (n = 8). The MTDR fluorescence intensity was normalized by the Hoechst 33342 (HO), and the results were expressed as a percentage related to shGFP. Bars represent the mean ± SD. ∗*p* < 0.05, ∗∗*p* < 0.01 and ∗∗∗*p* < 0.001 *versus* control condition. PABPC4, poly(A)–binding protein cytoplasmic 4.

PABPC1 is the most abundant cytoplasmic member of the PABPs family, and its expression is higher than PABPC4 in myotubes (Fig. 4*A*). Additionally, both PABPC1 and PABPC4 mRNA levels were decreased during cellular differentiation (Fig. 4*A*). To examine a potential compensatory effect between PABPC1 and PABPC4, we silenced either PABPC1 or PABPC4. Neither PABPC1 nor PABPC4 KD reduced the protein content of the other isoform (Fig. 4, *B*–*D*). Considering that the canonical role of PABPCs is the stabilization of RNA and protein translation, we also evaluated if the KD of each isoform affects protein translation. Only PABPC1 KD reduced protein translation (Fig. 4, *E* and *F*). To test if PABPC1 also regulates mitochondrial function, we evaluated the rate of cellular respiration. Unlike PABPC4 KD, PABPC1 KD did not change the OCR in myotubes compared to control cells (Fig. 4*G*). Consistent with this result, PABPC1 KD had no effect on lactate production neither in high glucose medium nor during galactose treatment (Fig. 4*H*). Moreover, the mitochondrial content was not increased in PABPC1 KD, as shown in PABPC4 KD cells in both high glucose and galactose medium (Fig. 4*I*). These findings suggest that PABPC4 alone, and not PABPC1, is a critical modulator of mitochondrial function and supports its potential involvement in mitochondrial regulation during metabolic stress in skeletal muscle cells.

### PABPC4 protein content is modulated in the same fashion as NCoR1

**Figure 5:**
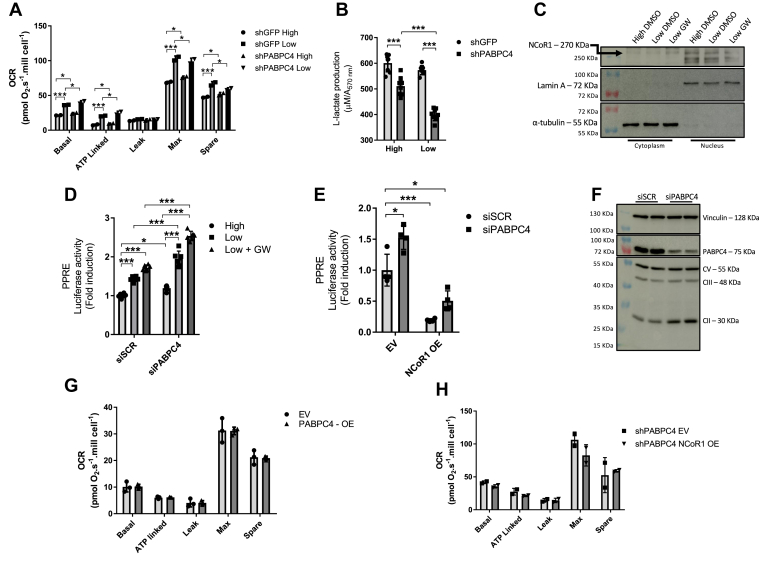
**PABPC4 knockdown increases PPARβ activity.***A*, either shGFP or shPABPC4 myotubes were seeded in 60 mm Petri dishes and maintained for 12 h in high (25 mM) or low (5 mM). Cells were then trypsinized, counted, and loaded on Oroboros chambers, and the oxygen consumption rate (OCR) was recorded (n = 2). *B*, lactate released into the media was measured after the cells were maintained in high (25 mM) or low (5 mM) glucose-containing media. *C*, C2C12 were seeded in 60 mm Petri dishes and differentiated into myotubes and maintained for 12 h in high (25 mM), low (5 mM), or low plus GW (100 mM GW501516, a PPARD agonist) medium (n = 4). Cells were then lysed, and nuclear and cytosolic fractions were isolated and then subjected to Western blotting to detect NCoR1 content (n = 1). *D*, MEF cells were seeded in 48-well plates, and when the confluency reached 50%, the cells were transiently transfected with non-target siRNA (siSCR) or siRNA specific to PABPC4 (siPABPC4). Twenty-four hours after siRNA transfection, cells were transiently transfected with PPAR response element (PPRE) and non inducible *Renilla* luciferase–expressing plasmids. Twenty-four hours after plasmid transfection, the medium was changed, and cells were maintained in a high (25 mM), low (5 mM), or low + GW (5 mM glucose + 100 mM GW501516) medium for 12 h, and the luminescence was recorded. *Firefly* luminescence was normalized by *Renilla* luminescence, and values expressed as fold change related to siSCR (n = 6). *E*, MEF cells were seeded in 48-well plates and transiently transfected with siRNA as described before. Twenty-four hours after siRNA transfection, cells were transiently transfected with PPRE and non inducible *Renilla* luciferase–expressing plasmids. Additionally, cells were also transiently transfected with pcDNA-GFP-FLAG (EV)– or pCMX-NCoR1-FLAG (NCoR1 OE)–expressing plasmids. Forty-eight hours after plasmid transfection, cells were lysed and both the *Firefly* and *Renilla* luminescences were recorded. *Firefly* luminescence was normalized by *Renilla* luminescence, and values expressed as fold change related to siSCR EV (n = 4). *F*, Western blotting for proteins of mitochondrial electron transport chain in differentiated C2C12 transiently transfected with siSCR or siPABPC4. CV: ATP5A; CIII: UQCRC2; CII: SDHB. *G*, MEF cells were transiently transfected with pcDNA-GFP-FLAG (EV)– or pCMV6-PABPC4-FLAG (PABPC4 OE)–expressing plasmids, and 48 h after transfections, OCR was evaluated (n = 2). *H*, either control (shGFP) or PABPC4 knockdown cells (shPABPC4) were transiently transfected with pcDNA-GFP-FLAG (EV)– or pCMX-NCoR1-FLAG (NCoR1 OE)–expressing plasmids and 48 h after transfections, subjected to OCR (n = 2). Bars represent the mean ± SD. ∗*p* < 0.05 and ∗∗∗*p* < 0.001 *versus* control. MEF, mouse embryonic fibroblastic; NCoR1, nuclear receptor corepressor 1; PPAR, peroxisome proliferator-activated receptor; PABPC4, poly(A)–binding protein cytoplasmic 4.

We observed that PABPC4 KD cells had an increased oxidative phosphorylation capacity, and this effect was more pronounced under low glucose treatment (Fig. 5*A*). In the same fashion, low glucose treatment decreased lactate production with a more significant effect on PABPC4 KD cells (Fig. 5*B*). Glucose metabolism is highly impacted by NCoR1 activity, and muscle-specific NCoR1 KO mice have a higher oxidative phosphorylation capacity than WT littermates (2). To explore NCoR1 regulation under these conditions, we measured NCoR1 abundance by WB and found that NCoR1 protein content was reduced in the nucleus under nutrient deprivation, and this effect was more pronounced when associated with the PPARβ agonist GW (Fig. 5*C*). This result was also observed using fluorescence microscopy, where the low glucose treatment decreased the NCoR1accumulation inside the nucleus (Fig. S4*A*). To further assess the functional relevance of NCoR1 downregulation, we measured PPAR activity by a luciferase reporter assay using a plasmid construct containing the PPAR response element (15). As expected, overexpression of Pgc1α, a PPAR coactivator, increased PPAR activity, while NCoR1 overexpression had the opposite effect (Fig. S4*B*). Treatments with both low glucose alone and with the PPAR agonist (GW501516) increased PPAR activity, with the effect being more pronounced in the PABPC4 KD cells (Fig. 5*D*). To further explore the relationship between PPAR activation and NCoR1 and PABPC4 abundance, we overexpressed NCoR1 in control and PABPC4 KD cells. The NCoR1 overexpression significantly decreased PPAR transactivation in both siSCR and siPABPC4, suggesting that PABPC4 is an important regulator of NCoR1 (Fig. 5*E*). In line with the reporter assay, during nutrient deprivation, NCoR1 interact to a less extent with PPAR (Fig. S4*C*), and as a consequence, the expression of PPAR target genes is upregulated (Fig. S4*D*). Similarly, the PABPC4 KD increased the protein content of the mitochondrial ETC (Fig. 5*F*). Then, we examined whether PABPC overexpression could affect the OCR. The overexpression of PABPC4 did not change the OCR (Fig. 5*G*). To test the hypothesis that the effect of PABPC4 KD on mitochondrial function is mediated by a lower NCoR1 content, we overexpressed NCoR1 in shPABPC4 cells. As shown in Figure 5*H*, NCoR1 overexpression in PABPC4 KD cells did not affect the OCR. Moreover, the interaction between PABPC4 and NCoR1 is modulated when cells are subjected to glucose deprivation (Fig. S4*E*), corroborating that nutrient availability increases PPAR activity by a lower interaction between PABPC4 and NCoR1.

### PABPC4 is necessary for NCoR1 stabilization

**Figure 6:**
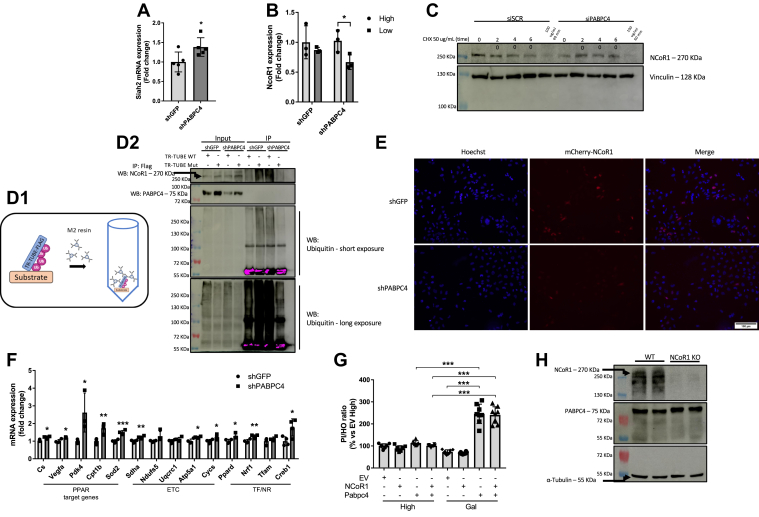
**PABPC4 is required for NCoR1 stability.***A*, Siah2 gene expression analysis was performed by RT-qPCR in either shGFP or shPABPC4 myotubes (n = 4). *B*, NCoR1 gene expression analysis was performed by RT-qPCR in shGFP or shPABPC4 myotubes maintained in high (25 mM) or low (5 mM) glucose DMEM, for 12 h (n = 3). *C*, NCoR1 protein half-life was analyzed in myotubes transiently transfected with siSCR or siPABPC4 by treating the cells with cycloheximide (CHX) followed by Western blotting (n = 1). CHX (50 or 100 μg/ml) was added at times indicated in the Figure. *D1*, trypsin-resistant tandem ubiquitin-binding entities (TR-TUBE) allow low-expressed and rapid-degraded proteins to be immunoprecipitated since it binds to ubiquitin molecules, stabilizing them and avoiding their degradation by the proteasome. Since the sequence is fused with a FLAG peptide, it allows proteins to be easily immunoprecipitated using an anti-FLAG antibody. *D2*, MEF cells were transiently transfected with WT TR-TUBE-FLAG (WT TR-TUBE) or mutated TR-TUBE-FLAG (Mut TR-TUBE). Cells were treated with 10 μM MG132 for 4 h and then subjected to immunoprecipitation using a FLAG antibody-bound resin as described in the Experimental procedures section (n = 1). Exposure time varied from short to long for capturing ubiquitinated proteins in the eluate and the input, respectively. *E*, MEFs stable GFP (shGFP) or PABPC4 (shPABPC4) knockdown were transiently transfected with pmCherry-NCoR1–expressing plasmid. Forty-eight hours after transfection, cells were fixed and observed in a fluorescence microscope. Nuclei were stained with Hoechst 33342. Magnification = 200 ×. *F*, gene expression performed by RT-qPCR in control (shGFP) or PABPC4 knockdown (shPABPC4) myotubes (n = 4). *G*, MEF cells were transiently transfected with pcDNA-GFP-FLAG (EV), pCMX-NCoR1-FLAG (NCoR1 OE), or pCMV6-PABPC4-FLAG–expressing plasmids, and 48 h after transfections, the assay of cellular viability was performed using propidium iodide (PI) and Hoechst 33342 (HO). Twelve hours before PI and HO addition, cells were maintained in DMEM containing either high glucose (25 mM) or galactose (10 mM). The fluorescence was read in 535/617 nm (Ex/Em) for PI and 350/461 nm (Ex/Em) for HO (n = 8). PI fluorescence was normalized by HO fluorescence, and values were expressed as a percentage related to EV high. *H*, Western blotting in WT or NCoR1 KO myotubes (n = 2). Bars represent the mean ± SD. ∗*p* < 0.05, ∗∗*p* < 0.01 and ∗∗∗*p* < 0.001 *versus* control. Atp5a1, ATP synthase F1 subunit alpha; Cpt1b, carnitine O-palmitoyltransferase 1; Creb1, cyclic AMP-responsive element-binding protein 1; CS, citrate synthase; Cycs, cytochrome c; DMEM, Dulbecco’s modified Eagle’s medium; ETC, electron transport chain; MEF, mouse embryonic fibroblastic; Nrf1, nuclear respiratory factor 1; Ndufs5, NADH:ubiquinone oxidoreductase subunit s5; NCoR1, nuclear receptor corepressor 1; Pdk4, pyruvate dehydrogenase (acetyl-transferring) kinase isozyme 4; Ppard, peroxisome proliferator-activated receptor delta; PABPC4, poly(A)–binding protein cytoplasmic 4; Sdha, succinate dehydrogenase (ubiquinone) flavoprotein subunit; Sod2, superoxide dismutase (Mn); Tfam, transcription factor A; TF/NR, transcription factor/nuclear receptor; TR, thyroid hormone receptor; TUBE, Tandem Ubiquitin Binding Entity; Vegfa, vascular endothelial growth factor A; Uqcrc1, ubiquinol-cytochrome c reductase core protein 1.

Once we had demonstrated that PABPC4 is important for NCoR1 activity, we asked whether NCoR1 abundance was influenced by PABPC4 KD. First, we assessed the effect of PABPC4 KD on the expression of Siah2, the ubiquitin E3 ligase responsible for tagging NCoR1 for degradation. The PABPC4 KD showed a trend to increase the Siah2 expression (Fig. 6*A*). We also analyzed NCoR1 expression in PABPC4 KD cells under high or low glucose availability, and we found that under normal glucose levels, there is no decrease in NCoR1 gene expression, whereas under low glucose concentration, NCoR1 gene expression was markedly reduced in PABPC4 KD cells (Fig. 6*B*). Considering that under low glucose levels, PABPC4 KD cells exhibit increased PPAR activity and mitochondrial respiration, we hypothesized that PABPC4 could act by stabilizing NCoR1 and preventing it from being degraded by the proteasome. To explore this possibility, we first performed a cycloheximide (CHX) chase experiment. Basal NCoR1 protein content was lower in PABPC4 KD cells, and the NCoR1 half-live was decreased in PABPC4 KD cells (Fig. 6*C*). To further explore the NCoR1 degradation process, we transfected the cells with a flag-tagged Tandem Ubiquitin Binding Entity (TUBE) and performed an immunoprecipitation assay in the presence of the proteasomal inhibit MG132. The abundance of ubiquitinated proteins in PABPC4 KD cells was increased compared to the control (Fig. 6*D*). Additionally, transfection of cells with NCoR1 fused with a fluorescent protein (mCherry) demonstrated that NCoR1 is more degraded in PABPC4 KD cells than in the control (Fig. 6*E*). In line with this hypothesis, the expression of PPAR target genes was increased in the PABPC4 KD cells compared with the control (Fig. 6*F*). To further explore the PABPC4 role in NCoR1 stabilization/degradation, we overexpressed NCoR1 and PABPC4 individually or together. The cells were submitted to treatment with galactose to force the cells to generate energy from oxidative phosphorylation. Under high glucose conditions, neither the overexpression of NCoR1 or PABPC4, nor both, changed their cellular viability. In contrast, when cells were exposed to galactose, PABPC4 overexpression was associated with the same reduction in viability as cells with PABPC4 and NCoR1 overexpression (Fig. 6*G*), indicating that PABPC4 is necessary for NCoR1 stabilization. To address whether NCoR1 might modulate PABPC4 protein content, NCoR1 was silenced in myotubes (NCoR1 KO). As expected, there was no change in PABPC4 protein content (Fig. 6*H*), corroborating our hypothesis that it is PABPC4 availability that regulates NCoR1 stabilization and not the opposite.

### Decreasing PABPC4 is important for lipid metabolism

**Figure 7:**
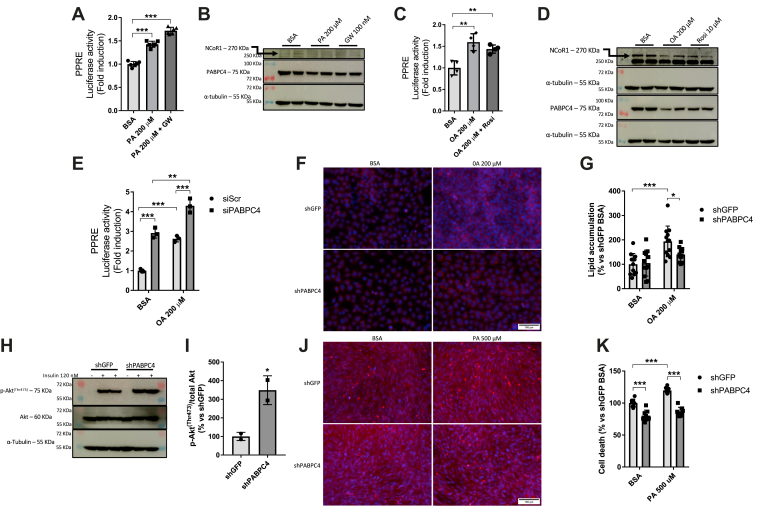
**Fatty acid metabolism leads to a lower expression of PABPC4 and NCoR1.***A*, MEF cells were seeded in 48-well plates and were transiently transfected with a plasmid expressing the PPAR response element (PPRE) and a non inducible *Renilla* luciferase and assayed 48 h later. Cells were treated for 12 h with 1% BSA, 200 μM palmitic acid (PA), or 200 μM PA + GW501516 (100 nM). *Firefly* luminescence was normalized by *Renilla* luminescence, and values were expressed as fold change related to BSA-treated cells (n = 4). *B*, Western blotting in myotubes with the same treatments as in (*A*) (n = 2). This experiment was repeated at least three times. *C*, MEF cells were transfected as described in (*A*) and treated for 12 h with 1% BSA, 200 μM oleic acid (OA), or 200 μM OA + 10 μM Rosiglitazone (Rosi). *D*, Western blotting in myotubes with the same treatments as in (*C*) (n = 2). This experiment was repeated at least three times. *E*, MEF cells were seeded in 48-well plates, and when the confluency reached 50%, the cells were transiently transfected with nontarget siRNA (siSCR) or siRNA specific to PABPC4 (siPABPC4). Twenty-four hours after siRNA transfection, cells were transiently transfected with plasmids expressing the PPRE and a non inducible *Renilla* luciferase. Twenty-four hours after plasmid transfection, the medium was changed, and cells were treated for 12 h with 1% BSA, 200 μM OA, or 200 μM OA + 10 μM Rosiglitazone (Rosi). *Firefly* luminescence was normalized by *Renilla* luminescence, and values expressed as fold change related to siSCR BSA (n = 4). *F*, lipid accumulation in control (shGFP) or PABPC4 knockdown (shPABPC4) MEF cells were seeded in 96-well plates. Lipid droplets were stained using LipidTOX (*red*), while nuclei were stained with Hoechst 33342 (*blue*) and visualized in a fluorescence microscope. Magnification = 200 ×. *G*, fluorescence from F was read at 577/609 nm (Ex/Em) for LipidTOX and 350/461 nm (Ex/Em) for Hoechst 33342. The LipidTOX fluorescence values were normalized by the Hoechst 33342 fluorescence values and expressed as a percentage related to shGFP BSA (n = 8). *H*, either shGFP or shPABPC4 C2C12 cells were differentiated and subjected to 2 h starving in KHB. Insulin (120 nM) was added for 20 min, and cells were then lysed in RIPA buffer and the lysates were subjected to Western blotting to assess the Akt phosphorylation. *I*, bar graph representing the quantitation from (*H*). *J*, cell death assay was performed using propidium iodide (PI) (*red*) and Hoechst 33342 (HO) (*blue*) in shGFP or shPABPC4 myotubes. Cells were seeded in 96-well plates and treated for 12 h with 1% BSA or 500 μM palmitic acid (PA), and cells were observed under a fluorescence microscope. *K*, samples from H had their fluorescence read at 535/617 nm (Ex/Em) for PI and at 350/461 nm (Ex/Em) for HO. PI fluorescence was normalized by HO fluorescence, and values were expressed as a percentage related to shGFP BSA (n = 8). Magnification = 200 ×. BSA, bovine serum albumin; MEF, mouse embryonic fibroblastic; NCoR1, nuclear receptor corepressor 1; PPAR, peroxisome proliferator-activated receptor; PABPC4, poly(A)–binding protein cytoplasmic 4.

As PABPC4 seems to play a role in NCoR1 stabilization, we hypothesized that in situations like lipid overload, when PPAR activity is increased, PABPC4 protein content would be downregulated. To test this hypothesis, we performed a reporter assay to assess the PPAR activity under free fatty acid (FFA) treatment in a concentration (200 μM) that is not toxic to the cells (Fig. S5*A*). As expected, the treatment with palmitic acid (PA) increased PPAR activity, as well as the treatment with PA along with a PPARβ agonist (Fig. 7*A*). Treatment with PA also decreased both the NCoR1 and PABPC4 protein content (Fig. 7*B*). Next, we performed the same experiments but using oleic acid (OA) instead. The treatment with OA increased the PPAR activity as well as the OA + PPARγ agonist rosiglitazone (Fig. 7*C*). In the same manner as PA treatment, OA treatment decreased both the NCoR1 and PABPC4 content (Fig. 7*D*). To assess whether PABPC4 plays a role in PPAR activation under FFA treatment, we treated the cells with OA. Indeed, the PABPC4 KD cells displayed increased PPAR activity compared to the control (Fig. 7*E*). In the shGFP control cells, exposure to OA led to the accumulation of intracellular lipid content, whereas this effect was abolished in shPABPC4 (Fig. 7, *F* and *G*).

Given the correlation between insulin response and mitochondrial capacity in skeletal muscle, we next investigated Akt phosphorylation levels to evaluate insulin sensitivity. In accord with the results observed upon FFA treatment, PABPC4 KD cells showed increased Akt phosphorylation when exposed to insulin (Fig. 7, *H* and *I*). We also asked whether PABPC4 KD could reduce cell death upon treatment with a toxic FFA concentration. As expected, the effect of lipid overload on cell death was decreased in PABPC4 KD cells (Fig. 7, *J* and *K*). To better evaluate the importance of PABPC4 regulation for lipid metabolism, we analyzed publicly available data and found a significant enrichment in terms related to mitochondrial and lipid metabolism in datasets in which PABPC4 was reduced (Fig. S5, *B* and *C*). Using the same approach, we analyzed the transcriptional signature of mice subjected to exercise training and found that terms related to mitochondrial and lipid metabolism were similarly enriched (Fig. S5*D*). Moreover, PABPC4 KO mice also exhibited lower plasmatic FFA and cholesterol concentrations (Fig. S5, *E* and *F*), as well as the respiratory exchange ratio, indicating that these mice oxidize more lipid than their WT littermates (Fig. S5*G*).

## Discussion

NCoR1 is ubiquitously expressed in different species and has been demonstrated to repress the transcriptional activity of nuclear receptors by a conserved mechanism (12). As such, knockout of NCoR1 in mice results in increased metabolic homeostasis and improved insulin response (13), two hallmarks linked to a lower prevalence of metabolic disorders (16, 17). However, the mechanism by which NCoR1 is regulated is still elusive. In this study, we immunoprecipitated NCoR1 and found PABPC4 as a new functional interactor of NCoR1. Pulldown analysis revealed that PABPC4 bind to the N-terminal region of NCoR1, whereas its C-terminal has been described to interact with transcription factors (18). Along with TBL1 and HDAC3, GPS2 and TBLR1 have been identified as NCoR1 interactors, comprising the NCoR1 complex (19). These mentioned proteins interact with NCoR1 at its n-terminus and modulate its activity (20). Either TBL1 or TBLR1 are proteins involved in the recruitment of proteasomal components and the degradation of NCoR1. This allows transcription factors to exchange from corepressors to coactivators and, consequently, promotes their transcriptional activity (19). In the same fashion, our findings demonstrate that the interaction of PABPC4 with the n-terminus region of NCoR1 is sufficient to avoid NCoR1 degradation. These findings, therefore, suggest that the NCoR1–PABPC4 interface might be an important metabolic target to induce NCoR1 derepression in skeletal muscle cells. In fact, our results reveal that PABPC4 works in a concerted action with NCoR1 in response to metabolic stress. Our results indicate that under several metabolic stressors, PABPC4 protein content is decreased, disrupting the interaction between PABPC4 and NCoR1 and leading to an increase in NCoR1 protein degradation by the proteasome and, consequently, increased metabolic activity. Notably, PABPC4 was found markedly reduced in C2C12 during the myogenesis process, in mice under caloric restriction, and in humans after exercise training. All these models are associated with increased mitochondrial biogenesis, suggesting that reduced expression of PABPC4 may be an adaptive event required to boost mitochondrial function in skeletal muscle cells. The Biogrid database analysis identified 18 common interactors for both PABPC4 and NCoR1. Most of these proteins were associated with response to estradiol or RNA metabolism, suggesting that the role of PABPC4 regulating NCoR1 and mitochondrial homeostasis may be targeted to protect cells against metabolic dysfunction. Estradiol is both the most abundant and most active estrogen in humans (21) and has important roles in cell proliferation and differentiation processes. Recent studies have shown that estradiol can bind to ERR (22, 23), a nuclear receptor well-known to induce mitochondrial biogenesis and oxidative phosphorylation capacity (24, 25).

PABPC4 silencing had a clear impact on the oxidative phenotype of C2C12 cells as demonstrated by increased mitochondrial respiration, citrate synthase activity, mtDNA content, and reduced lactate released to the extracellular medium. PABPC4 KD cells were also observed to exhibit improved insulin response and lower cell death, clearly indicating an augmented oxidative phenotype. This effect was further observed in MEF cells, thus suggesting that this mechanism might be present in different tissues where NCoR1 has a central effect over mitochondrial metabolism.

Given the role of PABPC1 in regulating translational processes and the fact that PABPC1-4 is essential for normal vertebrate development (26), it was surprising to find that reduced PABPC4 expression was associated with improved mitochondrial homeostasis in skeletal muscle cells. To examine a potential compensatory effect between PABPC1 and PABPC4, we knocked down either PABPC1 or PABPC4. Neither PABPC1 nor PABPC4 KD affected the protein content of the other, indicating that the silencing of PABPC4 was not compensated by PABPC1. Indeed, the nonredundant role of PABPs has already been demonstrated elsewhere (27). Moreover, we evaluated if the KD of each isoform had effect on protein translation. Only the KD of PABPC1 decreased the protein translation with no effect on OCR. The higher expression of PABPC1 and its lack of effect on mitochondrial function suggest that PABPC4 might have a critical role in muscular mitochondrial metabolism. Recently, Shan *et al*. (28) reported in an elegant paper that under metabolic stress, PABPC1 is retained in the nuclear compartment deactivating its poly(A) RNA binding and thus attenuating protein synthesis and, consequently, energy consumption.

NCoR1 degradation is linked to increased energy metabolism (12, 20). We found that the silencing of PABPC4 had a clear effect on PPAR transactivation. This effect was even higher when the cells were exposed to both low glucose and low glucose plus PPAR agonist GW501516. In contrast, the overexpression of NCoR1 in PABPC4 KD cells abolished this effect, indicating that the metabolic phenotype observed in PABPC4 KD cells is mediated by the reduction in NCoR1 stability. Consistent with these findings, the interaction between PABPC4 and NCoR1 was reduced when cells were subjected to glucose deprivation, suggesting that regulating the interaction between PABPC4 and NCoR1 is an important contributing factor to PPAR induction under physiological conditions. The observation that PABPC4 and NCoR1 work in a concerted action to induce PPARβ transactivation was confirmed in C2C12 cells–exposed lipid stress, with both PA and OA promoting PABPC4 and NCoR1 downregulation.

Mechanistically, we showed that PABPC4 is required for stabilizing NCoR1 and preventing its degradation by the proteasome. Experimental analysis evidenced that either the NCoR1 protein content or that of NCoR1 fused with mCherry was lower in the PABPC4 KD cells, whereas the abundance of ubiquitination associated with pulled-down NCoR1 was increased in PABPC4 KD cells compared to the control. Consistent with these findings, the expression of genes targeted by PPAR was markedly upregulated in the PABPC4 KD cells, suggesting that NCoR1 is regulated by PABPC4 through a ubiquitin-dependent mechanism.

**Figure 8:**
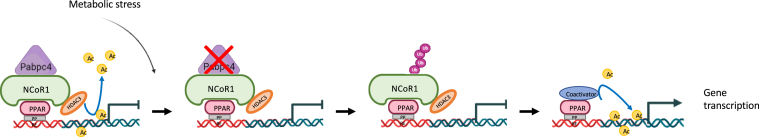
**Model depicting NCoR1–PABPC4 interaction during energetic stress.** The most accepted model of NCoR1 action is based on its binding partners, such as HDAC3, TBLR1, and GPS2. HADC3, specifically, is an enzyme responsible for removing acetyl (Ac) groups from chromatin proteins, hindering the access of the basal transcriptional machinery and, as a consequence, decreasing the gene transcription rate. According to our findings, PABPC4 interacts with NCoR1 when the cells are not under stressful conditions. However, under energetic stress, including low glucose, galactose, and chemical mitochondrial uncoupling treatment, PABPC4 levels are decreased. This leads to increased NCoR1 ubiquitination and subsequent degradation by the proteasome. Consequently, PPAR interacts with coactivators and can drive the transcription of genes necessary for cells to respond to stressful situations. NCoR1, nuclear receptor corepressor 1; PPAR, peroxisome proliferator-activated receptor; PABPC4, poly(A)–binding protein cytoplasmic 4.

Based on these findings, we propose a model where PABPC4 works in a concerted action with NCoR1 in response to metabolic stress (Fig. 8). Under such conditions, a decrease in PABPC4 content in skeletal muscle cells may be an adaptive event required to degrade NCoR1 and consequently induce mitochondrial metabolism by a PPARβ-dependent mechanism. Therefore, this new metabolic interface mediated by a physical interaction between PABPC4 and NCoR1 may provide new insights for targeting metabolic disorders.

## Experimental procedures

### Cell culture and treatments

C2C12, Hek293T, and MEF cells were cultivated in Dulbecco’s modified Eagle’s medium (DMEM) containing 10% fetal bovine serum (FBS) and penicillin (100 U/ml)/streptomycin (100 μg/ml) at 37 °C and 5% CO_2_. To induce C2C12 cells differentiation, the medium was replaced with DMEM containing horse serum (2%) and penicillin (100 U/ml)/streptomycin (100 μg/ml). The cells were kept for 5 days at 37 °C and 5% CO_2_. Cells were seeded in 0.2% gelatin-coated plates to avoid cellular detachment during differentiation. To induce energetic stressful condition, the growth medium containing high glucose concentration (25 mM) was switched to either low glucose medium (5 mM) or galactose medium (10 mM) without glucose for 12 h. Sodium pyruvate and L-glutamine concentration were kept at 1 mM and 4 mM, respectively. The mitochondrial stress was also induced with mitochondrial uncoupler carbonyl cyanide 3-chlorophenylhydrazone (10 μM) for 16 h. The treatment with PPARD agonist GW501516 (Sigma-Aldrich, 100 nM) or with PPARγ rosiglitazone (Sigma-Aldrich, 10 μM) was performed for 12 h.

### Animal procedures

Four-week-old male C57BL/6Junib mice were maintained in collective cages (4 animals/cage) at 22 °C in a 12 h light-dark cycle. The animals had free access to water and standard rodent chow diet (Nuvilab-CR1), and all experiments were in agreement with the rules for the scientific use of animals issued by the National Council of Control of Animal Experimentation (CONCEA) and approved by the Ethics Commission on Animal Use of the University of Campinas - CEUA/UNICAMP under the certificate number 5626-1/2020.

### Measurement of maximal oxygen consumption

At the end of the adaptation period, the mice were submitted to the measurement of maximal oxygen consumption (VO_2_max) test on a treadmill with a 25º inclination coupled to a gas analyzer system. The VO_2_max was recorded on METABOLISM software (https://panlab.com/en/products/metabolism-software-panlab) (Panlab/Harvard device) as published elsewhere (29).

### Acute physical exercise

The animals were placed on a treadmill for 5 days at 8 cm/s, for 10 min, with 25° inclination for adaptation. Between days six and seven, it was given a rest period to the animals, and on day 8, the animals underwent aerobic exercise at 70% of VO_2_max until exhaustion. Exhaustion was defined as the instant in which the mouse was unable to continue running even with stimulation by means of electric shocks at 1 mA (13). After the exercise, the animals were anaesthetized with ketamine (300 mg/kg) and xylazine (30 mg/kg), followed by cervical dislocation, and the red gastrocnemius muscles were collected and stored at −80 °C for further analysis.

### Plasmids construction

The pCMX-NCoR1-Flag plasmid was a kind gift from Dr Valentina Perissi (Boston University), and the coding sequence was subcloned into de pmCherry-C1 plasmid. Briefly, the DNA was amplified by PCR using primers with restriction sites to SalI at both ends. The amplicon was electrophoretically separated, and the band corresponding to NCoR1 was excised and purified from the gel using spin columns (QIAquick Gel Extraction Kit, Qiagen). Then, the amplicon was digested with SalI (Anza, Thermo Fisher Scientific), electrophoretically separated, and purified as described above. The insert was then ligated in the pmCherry-C1 vector using T4 DNA ligase (New England Biolabs) and transformed into *Escherichia coli* DH5-α. Bacteria were plated on 50 μg/ml kanamycin plates, and positive clones were screened by colony PCR. The plasmid was subjected to sequencing to ensure that no mutation was present in the plasmid DNA sequence and that it was in frame. The pLKO.1-puro plasmid (Addgene #8453) was used to produce a PABPC1 (NM_008774.3) KD cellular lineage. Briefly, the pLKO.1-puro plasmid was digested with AgeI and EcoRI (Anza, Thermo Fisher Scientific), and the annealed oligo (Table 1) was ligated using T4 DNA ligase (New England Biolabs). The target sequence (TRCN0000054952) was defined using the tool at the Broad Institute (https://portals.broadinstitute.org/gpp/public/). The ligation product was then used to transform Stbl3 *E. coli*, and the positive colonies were screened and subjected to sequencing. NCoR1 (NM_001252313.1) KD cells were produced using the plasmid pLKO.1-puro and the same cloning procedures described above. The target sequence (TRCN0000096474) was obtained using the tool from Broad Institute. PABPC4 (NM_130881.2) KD cells were produced using the plasmid pLKO.1-puro-shPABPC4 (Sigma-Aldrich, TRCN0000-102378) and the same cloning procedures described above. The lentiCRISPR V2 plasmid (Addgene #52961) was used to produce the NCoR1 KO cellular lineage. Briefly, the plasmid was digested using BmsBI (Anza, Thermo Fisher Scientific), and the annealed oligo (Table 1) was ligated as previously described. All the next cloning steps were performed as described in shPABPC1 cloning procedures. To insert the PABPC4 coding sequence into the empty pGEX-4T1 vector, the PABPC4 sequence containing both SalI and NotI restriction sites was amplified by PCR. The pGEX-4T1 vector was digested using SalI and NotI restriction enzymes (both from Anza, Thermo Fisher Scientific), and the PABPC4 sequence was inserted, generating a 2 kb fragment which contains the GST-tag sequence used for protein purification. The NCoR1 constructs were amplified by PCR using the pCMX-NCoR1-Flag plasmid as template. Both the 4.9 kb N-terminal fragment (N-NCoR1) and the 2.5 kb C-terminal fragment (C-NCoR1) were inserted into the plasmid pCMV6–Entry-FLAG (pCMV6–NCoR1 N-terminal-FLAG and pCMV6–NCoR1 C-terminal-FLAG), between the SfaAI and MluI (both from Anza, Thermo Fisher Scientific) restriction sites. All plasmids were subjected to sequencing to confirm that no mutation was introduced. All primer sequences are listed in Table 1.

**Table 1:** Primers used in this work

Usage	Primer name	Sequence (5′-3′)
Cloning primer	pLKO_shGFP sense	CCGGCAAGCTGACCCTGAAGTTCATCTCGAGATGAACTTCAGGGTCAGCTTGTTTTTG
Cloning primer	pLKO_shGFP antisense	AATTCAAAAACAAGCTGACCCTGAAGTTCATCTCGAGATGAACTTCAGGGTCAGCTTG
Cloning primer	pLKO_shPABPC4 sense (TRCN0000102378)	CCGGGCCAGTTTGGTAAGACCCTAACTCGAGTTAGGGTCTTACCAAACTGGCTTTTTG
Cloning primer	pLKO_shPABPC4 antisense (TRCN0000102378)	AATTCAAAAAGCCAGTTTGGTAAGACCCTAACTCGAGTTAGGGTCTTACCAAACTGGC
Cloning primer	pLKO_shPABPC1 sense (TRCN0000054952)	CCGGCCTAGCCAAATTGCTCAACTACTCGAGTAGTTGAGCAATTTGGCTAGGTTTTTG
Cloning primer	pLKO_shPABPC1 antisense (TRCN0000054952)	AATTCAAAAACCTAGCCAAATTGCTCAACTACTCGAGTAGTTGAGCAATTTGGCTAGG
Cloning primer	pLKO_shNCoR1 sense (TRCN0000096474)	CCGGCCTCTAATACAGGCACTTCAACTCGAGTTGAAGTGCCTGTATTAGAGGTTTTTG
Cloning primer	pLKO_shNCoR1 antisense (TRCN0000096474)	AATTCAAAAACCTCTAATACAGGCACTTCAACTCGAGTTGAAGTGCCTGTATTAGAGG
Cloning primer	lentiCRISPR NCoR Exon 11-1 sense	CACCGTCTGCATCAAACATCATAGG
Cloning primer	lentiCRISPR NCoR Exon 11-1 antisense	AAACCCTATGATGTTTGATGCAGAC
Cloning primer	NCoR1 SalI Fw	AAGTCGACGATGATGGACTTGGAATTGCC
Cloning primer	NCoR1 SalI Rv	AAGTCGACCTAGTTTTTCTTTGTATCTGGC
Cloning primer	pGEX4T1-PABPC4 Fw	GAGAGTCGACAAATGAACGCTGCAGCCAGCAG
Cloning primer	pGEX4T1-PABPC4 Rv	GAGAGCGGCCGCTAAAGAGGTAGCAGCAGCAACAG
Cloning primer	pCMV6-NCoR1- N-terminal Fw	GAGACCGCGATCGCATGGATTACAAGGATGACGACGATAAGTCAAGTTCAGGTTA
Cloning primer	pCMV6-NCoR1- N-terminal Rv	TCCACGCGTCTGCTGTGAGGTAATGTAATCGTTGAG
Cloning primer	pCMV6-NCoR1- C-terminal Fw	GAGACCGCGATCGCATGGATTACAAGGATGACGACGATAAGATGCAGGTGAATCTG
Cloning primer	pCMV6-NCoR1- C-terminal Rv	TCCACGCGTTCAGTCGTCACTATCAGA
qPCR primer	Rpl39 Fw	CAAAATCGCCCTATTCCTCA
qPCR primer	Rpl39 Rv	AGACCCAGCTTCGTTCTCCT
qPCR primer	Pabpc4 Fw	TGGTCTGTGATGAGAACGGC
qPCR primer	Pabpc4 Rv	ACCCACGAACACTTTACGGT
qPCR primer	Siah2_Fw	CCAATGCCGCCAGAAGTTAAG
qPCR primer	Siah2_Rv	CAGGGAAACAGAACTGCCGA
qPCR primer	PABPC1 Fw	GAATATGCCCGGTGCTATCCG
qPCR primer	PABPC1 Rv	ACTCGTGGAACCTGTGAGGAA
qPCR primer	Ncor1 Fw	CTGGTCTTTCAGCCACCATT
qPCR primer	Ncor1 Rv	CCTTCATTGGATCCTCCATC
qPCR primer	Bcs1l Fw	CTGAATGGCGTACCTTTGGT
qPCR primer	Bcs1l Rv	AGCCACGTCTGTAGGGAATG
qPCR primer	Atp5a1 Fw	ACTGCATCTACGTCGCGATT
qPCR primer	Atp5a1 Rv	CGCATCCGTCAGTCTCTTCA
qPCR primer	Ucp3 Fw	ATGAGTTTTGCCTCCATTCG
qPCR primer	Ucp3 Rv	GGCGTATCATGGCTTGAAAT
qPCR primer	Cs Fw	GTGTCAGATGAGAAGTTACGAGA
qPCR primer	Cs Rv	TCCTTAGGCAGATGTTTCAG
qPCR primer	Vegfa Fw	GCACATAGAGAGAATGAGCTTCC
qPCR primer	Vegfa Rv	CTCCGCTCTGAACAAGGCT
qPCR primer	Pdk4 Fw	GGATTACTGACCGCCTCTTTAG
qPCR primer	Pdk4 Rv	GTAACCAAAACCAGCCAAAGG
qPCR primer	Cpt1b Fw	CCTGGTGCTCAAGTCATGGT
qPCR primer	Cpt1b Rv	TCCTGCTTCGGAGGTAGACA
qPCR primer	Sod2 Fw	ACTGAAGTTCAATGGTGGGG
qPCR primer	Sod Rv	GCTTGATAGCCTCCAGCAAC
qPCR primer	Sdha Fw	GGAACACTCCAAAAACAGACCT
qPCR primer	Sdha Rv	CCACCACTGGGTATTGAGTAGAA
qPCR primer	Ndufs5 Fw	GACATACAGAAAAAGCTGGGCA
qPCR primer	Ndufs5 Rv	TCGCCTCATCGTTTTGTACCG
qPCR primer	Uqcrc1 Fw	GGCCGATCTGCTGTTTCAG
qPCR primer	Uqcrc1 Rv	CATCTCGCATTAACCCCAGTT
qPCR primer	Cycs Fw	AGGCAAGCATAAGACTGGAC
qPCR primer	Cycs Rv	ACTCCATCAGGGTATCCTCTC
qPCR primer	Ppargc1a Fw	TGAACGCACCTTAAGTGTGGAA
qPCR primer	Ppargc1a Rv	GGGTTATCTTGGTTGGCTTTATGA
qPCR primer	Nrf1 Fw	TGCCCAAGTGAATTACTCTGC
qPCR primer	Nrf1 Rv	TCGTCTGGATGGTCATTTCAC3
qPCR primer	Tfam Fw	CACCCAGATGCAAAACTTTCAG
qPCR primer	Tfam Rv	CTGCTCTTTATACTTGCTCACAG
qPCR primer	Creb1 Fw	CTTGGTGCTGGGCACTAGA
qPCR primer	Creb1 Rv	ACCCCGATTACCAAACTAGC
qPCR primer	Hadh Fw	TCTTGACTATGTTGGACTGGATAC
qPCR primer	Hadh Rv	AAGGACTGGGCTGAAATAAGG
qPCR primer	Alas1 Fw	TCGCCGATGCCCATTCTTATC
qPCR primer	Alas1 Rv	GGCCCCAACTTCCATCATCT
semiquantitative PCR	Mtnd1 Fw	CCCATTCGCGTTATTCTT
semiquantitative PCR	Mtnd1 Rv	AAGTTGATCGTAACGGAAGC
semiquantitative PCR	LPL Fw	GGATGGACGGTAAGAGTGATTC
semiquantitative PCR	LPL Rv	ATCCAAGGGTAGCAGACAGGT

### Generation of lentiviral vectors and establishment of genetically modified cells

The viral preparations were produced and tittered by the Viral Vector Laboratory at CNPEM, as previously described (30). The shRNA-expressing cells were established by virus transduction using a multiplicity of infection of 10, the presence of 8 μg/ml of polybrene. The selection was performed in 2 μg/ml puromycin-supplemented growth medium. The shRNA-expressing cells were used as a pool, while in the cells expressing the sgRNA, a single colony was selected.

### Transient plasmid transfection

Hek293T and MEF cells were transfected at 70% of confluency in Opti-MEM medium (Thermo Fisher Scientific) using PEI at 1:3 DNA:PEI ratio. Cells were harvested 48 h after transfection.

### siRNA transfection

MEF cells were transfected with specific siRNA oligos against the PABPC4 gene (SASI_Mm0100196797_AS, Sigma-Aldrich) or nontarget siRNA as a control (AllStars Negative Control siRNA, Qiagen). The cells were seeded in 48-well plates, and when confluency reached 50%, the medium was changed to 250 μl of OPTIMEM medium containing 20 pmol nontarget control siRNA or specific siRNA and Lipofectamine RNAiMAX (5 μl) (Life Technologies). The experimental assays were carried out 72 h after transfection. For C2C12 cells, siRNA transfection was performed in the differentiation medium, and the other conditions were kept as described for MEF cells and assayed 72 h after transfection.

### PABPC4-GST overexpression and cellular lysis for pull-down assays

Large-scale expression of pGEX4T1-PABPC4 and pGEX4T1-GST was performed in chemically competent *E. coli* BL21 (DE3) Star. Protein expression was induced with 1 mM IPTG for 5 h, at 27 °C, or for 2 h, at 37 °C. Bacteria were centrifuged at 3000*g*, for 5 min, 4 °C, and the pellet was resuspended in 0.5 ml of lysis buffer containing 50 mM Tris–HCl, pH 8.0, 150 mM NaCl, 0.5% Triton-X100, 0.15 mM PMSF, 2 μg/ml aprotinin, 0.1 mg/ml lysozyme, 1× protease cocktail (SigmaFast, Sigma-Aldrich), and 5% glycerol. Then, the resuspended pellet was incubated for 1 h in a shaker at 4 °C and sonicated 8 times for 15 s, at 20% amplitude, with 2-min intervals between pulses. The samples were centrifuged at 20,000*g* for 15 min at 4 °C. The supernatant was collected fresh for pull-down, and the pellet was discarded.

### Overexpression of NCoR1 full-length, N-NCoR1 and C-NCoR1 fractions and cellular lysis

To overexpress NCoR1 full-length, N-NCoR1 and C-NCoR1 constructs, Hek293T cells were used for ease of transfection and gene manipulation. The cells were plated in 100 mm Petri dishes in DMEM (10% FBS) and incubated in atmosphere containing CO_2_ (5%) at 37 ºC until reaching 70% of confluence. The cells were transfected with 10 μg of plasmidial DNA using PEI (1:3 DNA:PEI ratio) in 500 μl of Opti-MEM medium (Thermo Fisher Scientific). The cells were harvested 48 h after transfection, and the transfection efficiency was assessed using a plasmid containing GFP (pcDNA-GFP-FLAG) under the same experimental conditions. The plates were kept at 37 °C with the transfection mixture for 16 h. Then, the medium was aspirated, and 8 ml of DMEM containing 10% FBS was added. The cells were expanded for 48 h, and the medium was changed when necessary. For collection and lysis, the DMEM medium was aspirated, and the plates washed with 1× PBS and then collected by scraping the plates with 1 ml of PBS. The cells were centrifuged at 500*g*, for 10 min, and the pellets were resuspended in 500 μl of NP-40 lysis buffer containing 50 mM Tris–HCl, pH 8.0, 150 mM NaCl, 0.1 mM EDTA, 1% NP-40, 0.1 mM PMSF, 0.1 mM sodium orthovanadate (Na_3_VO_4_), 5% glycerol, and supplemented with protease cocktail (SigmaFast, Sigma-Aldrich). Tubes were placed on ice for 30 min and then sonicated for 10 s at 30% amplitude and centrifuged at 20,000*g*, at 4 °C, for 20 min. Supernatants were either added to the resin for the pull-down assay or stored at −20 °C.

### Pull-down assay

Pull-down were performed with glutathione agarose resin using the “Batch Method” for protein purification as determined by the manufacturer's protocol (Thermo Fisher Scientific, #16100). To detect the protein interaction, fresh supernatant from lysed Star cells was added to 50 μl of resin (slurry) and kept under agitation at 4 °C for 16 h. After that, 668 μg of proteins were added to the resin in a ratio of 1:3 (protein/resin), a mass smaller than the resin's binding and saturation capacity. After binding of bait protein to the resin, the sample was centrifuged at 700*g*, for 2 min. The supernatant containing the unbound proteins was collected for Western blotting analysis in SDS-PAGE gel. Then, the resin was washed four times with buffer according to the manufacturer's protocol and incubated under agitation at 4 °C for 16 h, with Hek293T cellular lysate containing overexpressed proteins. The washing steps were repeated, as previously described. Once protein–protein interaction complexes were formed, they were eluted by adding a buffer containing 10 mM of reduced glutathione in two different steps, with volumes of 100 μl and 50 μl. For elution, the resin was incubated under agitation for 30 min, at room temperature. The samples were collected by centrifugation at 700*g*, for 2 min, at 4 °C and stored at −20 °C until visualization of proteins by Western blotting. It was applied 10% of Star bacterial cells lysate and 200 μg of protein from Hek293T cells lysate. About 10% of the fractions not bound to the resin (flow-through) and after the washing step were also applied in SDS-PAGE gel. Finally, one-third of the elution volume was used for the analysis of NCoR1 by SDS-PAGE.

### FFAs preparation

Cells were treated with PA or OA for 12 h at the concentrations described in the figure legends. The FFAs’ stock solutions were prepared firstly diluting either PA or OA in 95% ethanol to form a 50 mM solution and then conjugated with 1% bovine serum albumin (BSA) in DMEM at 40 °C, as described elsewhere (16).

### Coimmunoprecipitation

MEF cells were seeded in 100 mm Petri dishes and transfected with the plasmids as indicated in the figure legends. Cells were harvested 48 h after transfection using lysis buffer (25 mM Tris, pH 7.9, 150 mM NaCl, 1 mM EDTA, 1% Triton X-100) supplemented with 1 mM sodium fluoride (NaF), 1 mM Na_3_VO_4_, and protease inhibitor (SigmaFast, Sigma-Aldrich) and incubated on ice for 30 min. Cells were centrifuged at 14,000*g*, for 15 min, 4 °C. Protein concentration was measured using a colorimetric method (31), and about 1 to 2 mg of protein was used in each co-IP experiment. For NCoR1 immunoprecipitation, 4 μg of anti-NCoR1 antibody (Abcam, ab3482) or 4 μg of anti-Rabbit IgG as an internal control was added. Samples were incubated with Protein A/G Magnetic beads (Pierce #88803) overnight, at 4 °C, under rotation. The resin was washed in lysis buffer, the bound proteins were eluted in 0.1 M glycine buffer, pH 2.2, and samples were prepared accordingly for mass spectrometry experiment. PABPC4 was immunoprecipitated with anti-FLAG M2 affinity gel (40 μl) (Sigma-Aldrich), and the samples were incubated overnight, at 4 °C, under rotation. The resin was washed and the proteins bound were eluted with 150 ng/μl FLAG peptide (Sigma-Aldrich) in 10 mM Tris, pH 7.9, 300 mM NaCl, 1 mM EDTA, 0.05% SDS, 1% Triton X-100 supplemented with 1 mM NaF, 1 mM Na3VO, and protease inhibitor (SigmaFast, Sigma-Aldrich) or boiled with 2× SDS-PAGE buffer (32). To immunoprecipitate WT TR-TUBE-Flag and Mutated TR-TUBE-Flag, cells were treated for 4 h with the proteasomal inhibitor MG132 (10 μM). Both WT TR-TUBE-Flag and Mutated TR-TUBE-Flag plasmids were kindly donated by Dr Yukiko Yoshida (Tokyo Metropolitan Institute of Medical Science). The immunoprecipitation assay was performed as described for PABPC4.

### Mass spectrometry

Co-IP samples were treated with urea (8 M), followed by protein reduction with DTT (5 mM for 25 min at 56 °C) and alkylation with iodoacetamide (14 mM for 30 min at room temperature in the dark). For protein digestion, urea was diluted to a final concentration of 1.6 M, and calcium chloride (1 mM) was added for trypsin digestion during 16 h at 37 °C (1 μg of trypsin) as described by Villén and Gygi (33). The peptides derived from the trypsin-digested samples were desalted by stage-tips (34), dried in a vacuum concentrator, and reconstituted in formic acid (0.1%). An aliquot of 4.5 μl (∼2 μg peptides) of whole MEF extracts and 3.0 μl of NCoR1 immunoprecipitation were analyzed on an ETD enabled Orbitrap Velos mass spectrometer (Thermo Fisher Scientific) connected to the EASY-nLC system (Proxeon Biosystem) through a Proxeon nanoelectrospray ion source. Peptides from MEF extracts and NCoR1 immunoprecipitation were separated by a 2 to 30% acetonitrile gradient in 0.1% formic acid using an analytical column PicoFrit Column (20 cm x ID75 μm, 5 μm particle size, New Objective) at a flow rate of 300 nl/min over 173 min and 30 min, respectively. The nanoelectrospray voltage was set to 2.2 kV, and the source temperature was 275 °C. All instrument methods were set up in the data-dependent acquisition mode. The full scan MS spectra (m/z 300–1600) were acquired in the Orbitrap analyzer after accumulation to a target value of 1 × 10^6^. The resolution in the Orbitrap was set to r = 60,000, and the 20 most intense peptide ions with charge states ≥2 were sequentially isolated to a target value of 5000 and fragmented in the linear ion trap using low-energy CID (normalized collision energy of 35%). The signal threshold for triggering an MS/MS event was set to 1000 counts. Dynamic exclusion was enabled with an exclusion size list of 500, exclusion duration of 60 s, and a repeat count of 1. Activation q = 0.25 and activation time of 10 ms were used (35).

### Raw LC–MS/MS data analysis

The identification of proteins was performed with MaxQuant v.1.5 (27) against the Mouse Uniprot SwissProt (release June, 2018, 61,204 sequences, 27,622,288 residues) using the Andromeda search engine (36). As search parameters, a tolerance of 6 ppm was considered for precursor ions (MS search) and 0.5 Da for fragment ions (MS/MS search), with a maximum of two missed cleavages. Carbamidomethylation of cysteine was considered as fixed modification and oxidation of methionine and protein N-terminal acetylation as variable modifications. A maximum of 1% of false discovery rate was set for both protein and peptide identification. Protein quantification was performed using the label-free quantification (LFQ) algorithm implemented in MaxQuant environment, with one minimal ratio count and a 2 min window for matching between runs. Statistical analysis was performed with Perseus v1.5 software (https://maxquant.net/perseus/) (37), available at MaxQuant package. First, reverse and only identified by site entries were excluded from further analysis. LFQ was performed using the spectral protein intensities (LFQ). Exclusive and common proteins are represented as Venn diagram performed by InteractiVenn tool (38). For the analyses of differentially expressed proteins, minimum of two valid values in each group were considered and the *t* test was applied. The mass spectrometry proteomics data generated in this study are available at ProteomeXchange *via* the PRIDE partner repository (39) with the dataset identifier PXD040945.

### Oxygen consumption

Cells were trypsinized and centrifuged at 500*g*, 25 °C for 5 min. The cells were then resuspended in 1 ml of PBS containing and counted. The oxygen consumption was monitored after the sequential additions of 1 μM oligomycin, a step-wise addition of 1 μM carbonyl cyanide 3-chlorophenylhydrazone, and 1 μM antimycin A. The OCR was recorded through Oroboros Oxygraph-2K, and DatLab software package (OROBOROS, Innsbruck) was used for data acquisition (2-sec-time intervals) and analysis.

### Whole cell extract and subcellular fractionation

For whole-cell extract, cells were lysed in RIPA buffer (25 mM Tris, pH 7.4, 150 mM NaCl, 1% NP-40, 0.5% sodium deoxycholate, 0.1% SDS), supplemented with 10 mM NaF, 10 mM Na_3_VO_4_, 1 mM PMSF, and protease inhibitor cocktail (Complete, Roche) using an ultrasonic processor (for 10 s, at 30% of amplitude; Sonics VCX 750 Vibra Cell). For subcellular fractionation, about 1 × 10^5^ cells were seeded in 60 mm Petri dishes, and then the cytosolic proteins were extracted adding buffer 1 (10 mM Hepes, pH 7.9, 50 mM NaCl, 0.5 M sucrose, 0.1 mM EDTA, 0.5% Triton X-100) and supplemented with 0.1 mM DTT, 10 mM NaF, 10 mM NA_3_VO_4_, 1 mM PMSF, and protease inhibitor (SigmaFast, Sigma-Aldrich) and incubating the cells on ice for 15 min. The cells were centrifuged at 800*g*, for 10 min, 4 °C. The supernatants were collected and considered as the cytosolic fraction. After that, the pellets were washed with buffer 2 (with 10 mM Hepes, pH 7.9, 10 mM KCl, 0.1 mM EDTA, and 0.1 mM EGTA) supplemented with 0.1 mM DTT, 10 mM NaF, 10 mM Na_3_VO_4_, 1 mM PMSF, and protease inhibitor and centrifuged at 800*g*, for 10 min, 4 °C. The supernatant was removed, RIPA buffer was added to the pellets, and samples were sonicated and centrifuged as described for the whole cell lysate. The supernatants were collected and considered as the nuclear fraction. Protein concentration was estimated by a colorimetric assay (31).

### Western blotting

Protein samples were prepared in reduced and denatured forms (32) and resolved using SDS-PAGE. The proteins were transferred to a nitrocellulose membrane and then blocked with 5% nonfat milk in TBS-T (0.02 M Tris–HCl, 0.16 M NaCl, and 0.1% Tween-20, pH 7.4), at room temperature, for 1 h. The membranes were incubated overnight with primary antibodies PABPC4 (Bethyl, #A301-466A), NCoR1 (Affinity, #AF0270), PABPC1 (Thermo Fisher Scientific, #PA5-29883), FLAG (Sigma-Aldrich, #F1804), α-tubulin (Sigma-Aldrich, #T9026), OXPHOS (proteins of mitochondrial ETC) (Abcam, #ab110413), PPARD (Thermo Fisher Scientific, #PA1-823A), eIF4G (Cell Signaling Technology, #2498), puromycin (Merck, #MABE343), Vinculin (Cell Signaling Technology, #4650), Lamin A (Santa Cruz Biotechnology, #sc-71481), and β actin (Santa Cruz Biotechnology, #81178), Akt (Cell Signaling Technology, #9272), p-Akt^Thr308^ (Cell Signaling Technology, #9275), ubiquitin (Abcam, #ab7254), and GST (Sigma-Aldrich, #G7781). The membrane was then washed with TBS-T and incubated with horseradish peroxidase–conjugated secondary antibody (1:10,000) in TBS-T solution containing 5% nonfat for 1 h. Membranes were washed with TBS-T and then added the peroxidase substrate SuperSignal West Plus (Thermo Fisher Scientific), and the band intensities were captured in the ImageQuant LAS500 (GE Healthcare).

### CHX chase assay

Myotubes were treated with 50 μg/ml CHX for 20, 40, or 60 min, the medium was removed, and cells were washed with 1× PBS. The cells were lysed in RIPA as previously described, and protein samples were subjected to SDS-PAGE and Western blotting (40).

### Protein synthesis by surface sensing of translation

Cells were seeded in 12-well plates and when indicated in the Figure, the cells were pretreated with 30 μg/ml CHX (Sigma-Aldrich) for 30 min, at 37 °C. Then, the medium was replaced with normal growth medium containing 10 μg/ml puromycin (Sigma-Aldrich) for an additional 30 min at 37 °C. The medium was then removed, and the cells were washed with PBS and lysed in RIPA as previously described. Protein samples (20 μg) were separated using SDS-PAGE, and the amount of puromycin-labeled protein was identified by Western blotting (41).

### mRNA extraction and gene expression

RNA was extracted using Trizol (Thermo Fisher Scientific) according to the manufacturer’s instruction, and the RNA quantification was performed by spectrophotometry (260/280 nm). The cDNA synthesis was performed from total RNA (1 μg) using the High-Capacity cDNA Reverse Transcription Kit (Thermo Fisher Scientific) accordingly to the manufacturer’s instructions. Gene expression was quantified by RT-qPCR, and the reactions were carried out using 25 ng of cDNA and SYBR Green PCR Master mix (Invitrogen). The values were expressed as fold change (42). PCR primer sequences are listed in Table 1.

### Lactate concentration

The medium was removed, and the cells incubated with KHB buffer (118 mM NaCl, 4.7 mM KCl, 1.2 mM MgSO_4_, 1,25 CaCl_2_, 1.2 mM KH_2_PO_4_, 25 mM NaHCO_3_) supplemented with 25 mM glucose at 37 °C for 2 h. The KHB was collected, and the lactate concentration was determined using lactate dehydrogenase enzyme (1 μg/0.2 ml) in 100 mM Tris–HCl, pH 7.2, and 15 mM NAD^+^. NADH fluorescence was determined with the excitation wavelength of 360 and emission wavelength of 460 nm (43).

### Citrate synthase

Citrate synthase activity was assayed as described elsewhere (44). Briefly, the cells were collected in 0.175 M KCl, 2 mM EDTA, pH 7.4, sonicated for 10 s, at 30% of amplitude, and centrifuged at 16,000*g*, for 15 min, 4 °C. The supernatant was collected, and the protein concentration was determined as previously described. Approximately 10 μg of protein were added to a 96-well plate, and then the reaction mixture containing Tris, pH 8.3, 5,5′-dithiobis(2-nitrobenzoic acid), and acetyl-CoA was added. The absorbance was read at 412 nm before the addition of oxaloacetate and subtracted from the absorbance after the addition of oxaloacetate. After the addition of 10 mM oxaloacetate, the absorbance was recorded during 10 min. The citrate synthase activity was calculated using the difference (delta) between the last recorded absorbance within the linear portion of the curve.

### Mitochondria staining

Cells were plated in clear bottom 96-well plates (μClear, Greiner) and treated as described in the legend of the figure. The cells were then incubated with 100 nM MitoTracker Deep Red FM (Thermo Fisher Scientific) for 30 min, at 37 °C. The medium was removed, and 1 μg/ml Hoechst 33342 was added for 15 min, at 37 °C. The solution was removed and cells were washed with PBS and fixed with 4% neutral-buffered formalin for 10 min, at room temperature. The MitoTracker Deep Red FM fluorescence signal was acquired at 644/665 nm (Ex/Em) and 350/461 nm (Ex/Em) for Hoechst 33342. The mitochondria staining was visualized in a fluorescence microscope at the same wavelengths.

### Lipid staining

Cells were plated in clear bottom 96-well plates (μClear, Greiner) and treated with FFAs or BSA as described in the legend of the figure, and after 12 h, the cells were fixed in 4% neutral-buffered formalin, for 30 min, at room temperature. The fixative was removed, and cells were washed twice and then added PBS solution containing LipidTOX Neutral Lipid Stain (Thermo Fisher Scientific). The cells were incubated for 30 min at room temperature, the staining solution removed, and a solution containing Hoechst 33342 (1 μg/ml) (Thermo Fisher Scientific) was added for 20 min, at room temperature. The solution was removed, and the cells were washed twice in PBS, and the fluorescence signal was acquired at 577/609 nm (Ex/Em) for LipidTOX and 350/461 nm (Ex/Em) for Hoechst 33342. The lipid accumulation was the ratio between the lipidTOX and Hoechst 33342 fluorescence and expressed as a percentage related to control.

### Immunofluorescence

Cells were grown in glass coverslips and treated as described in the legend of the figure. The media was removed, and the cells were washed twice with PBS. Cells were fixed with 4% paraformaldehyde in PBS, for 10 min, at room temperature and permeabilized with 0.1% of Triton X-1000 in PBS for 10 min, at room temperature. A blocking solution containing 3% BSA in PBS was added for 30 min, at room temperature, and the primary antibodies were diluted in 3% BSA, 0.1% Tween-20 in PBS (PABPC4, Bethyl, #A301-466A, 1:400 dilution; NCoR1, Affinity, #AF0270, 1:250 dilution). Samples were incubated overnight, at 4 °C, in a humidified chamber. The cells were washed three times with PBS and incubated with fluorescent-conjugated secondary antibody for 1 h, at room temperature, in the dark. The nucleus was stained with 1 μg/ml of Hoechst 33342 for 15 min, at room temperature, in the dark. The coverslips were then washed with PBS and mounted on glass slides using a hardening mounting medium (Dako).

### Mitochondrial DNA copy number

C2C12 cells were seeded in 12-well plates and differentiated into myotubes as previously described. Cells were lysed in 100 μl of 0.5% SDS and sonicated for 1 s, at 25% amplitude. The DNA was purified using the phenol-chloroform method, and the DNA samples were used in PCR to assess the ratio of mitochondrial DNA related to nuclear DNA. The gene used as a mitochondrial marker was ND1, while the nuclear marker used was LPL (primer sequences are listed Table 1).

### Luciferase assay

Luciferase assays were performed in MEF cells platted in 24-well plates. Cells at 80% of confluence were transfected using PEI as previously described, with 250 ng of pPPRE X3-TK-luc (Addgene, #1015) and 25 ng of pRL-SV40 (Promega, #E2231). After 48 h, the cells were lysed with 100 μl of 1 × passive lysis buffer (Promega, #E194A), and luciferase activity was measured using 20 μl of lysate in a 96-well white plate (Costar) using the Dual luciferase assay system (Promega) according to the manufacturer's instructions. The luminescence emitted from *Renilla* (pRL-SV40) was used as internal control.

### Insulin signaling

Myotubes were starved for 2 h in KHB before the treatment with 120 nM insulin (Humalog, Lilly) in KHB, for 10 min. Then, cells were washed in ice-cold PBS and lysed in RIPA.

### Cell death assay

Approximately 1 × 10^3^ cells were seeded in 96-well plates and differentiated to myotubes as previously described. Cells were then treated with 1% BSA (control) or 500 μM PA for 12 h, at 37 °C. Then, 5 μg/ml propidium iodide (Thermo Fisher Scientific) was added to the medium, and the cells were incubated for 20 min at 37 °C. Then, 1 μg/ml Hoechst 33342 (Thermo Fisher Scientific) was added for an additional 20 min, at 37 °C. The medium was removed, and the cells were washed twice with PBS. The fluorescence was read in PBS with the wavelengths set to 535/617 nm (Ex/Em) for propidium iodide and 350/461 nm (Ex/Em) for Hoechst 33342. The cell death was set as the ratio between the propidium iodide and Hoechst 33342 fluorescence and expressed as a percentage related to control.

### *In silico* analysis

The String database (https://string-db.org/) was used to identify the interactions among proteins identified in the co-IP/mass spectrometry experiment. Publicly available datasets (GSE99963, GSE60591, GSE6323, and GSE128651) were retrieved from GEO datasets. The pathway enrichment analysis was performed in ShinyGO 0.76 (http://bioinformatics.sdstate.edu/go/) or David database (https://david.ncifcrf.gov/) and only pathways with a p-adjusted value lower than 0.05 and fold enrichment greater than 1 was considered. Pathway enrichment analysis was also analyzed using Enrichr database (https://maayanlab.cloud/Enrichr/), and the BioPlanet 2019 library and only pathways with a p-adjusted value lower than 0.05 was considered significant. Data from shPABPC4 in HepG2 cell were retrieved from ENCODE project website (https://www.encodeproject.org/) under the accession number ENCSR455VZH. It was used data from an experiment already performed and made available under the accession ENCFF288ZLD in which the nonsignificant results were excluded and the differentially expressed genes were sorted by the Log2FC. The gene list was analyzed on David database, as previously mentioned. The gene expression data in mice subjected to exercise training were retrieved from GEO database (accession number GSE128651). Raw data were reanalyzed, the quality of the sequencing was assessed using the FastQC tool, and the adapters were trimmed out. Reads were aligned using the genome GRCm39 as a reference and using the STAR tool. The differentially expressed genes were organized using the RStudio and DESeq2 tools, and genes with a padj < 0.05 considered as significant. Data from PABPC4 KO mice were retrieved from the international mouse phenotype consortium (2385206" title = "https://www.mousephenotype.org/data/genes/MGI:2385206">https://www.mousephenotype.org/data/genes/MGI:2385206).

### Statistical analyses

Data were analyzed in GraphPad Prism v7.0 and expressed as mean ± SD. Unless specified in the figure legend, the means were compared using one-way ANOVA followed by Bonferroni test when comparison was performed in more than two groups or Student’s *t* test when comparison was made between two groups. A significance level of *p* < 0.05 was used (45).

## Data availability

Mass spectrometry data are deposited to the ProteomeXchange Consortium *via* the PRIDE partner repository with the dataset identifier PXD040945. The data that support the finding described in this work are available from corresponding author upon request.

## Supporting information

This article contains supporting information.

## Conflict of interest

The authors declare that they have no conflicts of interest with the contents of this article.

## Supporting information

Suppl Figure 1

Suppl Figure 2

Suppl Figure 3

Suppl Figure 4

Suppl Figure 5

Suppl Figure 5 CONTINUED
