# Supplementary figures and images for: Interaction between poly(A)–binding protein PABPC4 and nuclear receptor corepressor NCoR1 modulates a metabolic stress response
Source: J Biol Chem. 2023 Apr 12;299(6):104702. doi: 10.1016/j.jbc.2023.104702 (PMC10203745; doi:10.1016/j.jbc.2023.104702)

# Suppl. Figure 1

**A**

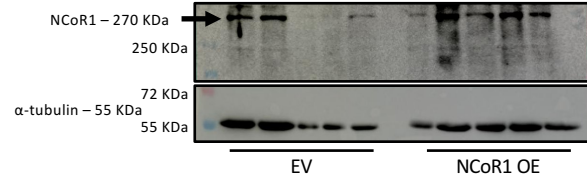

**B**

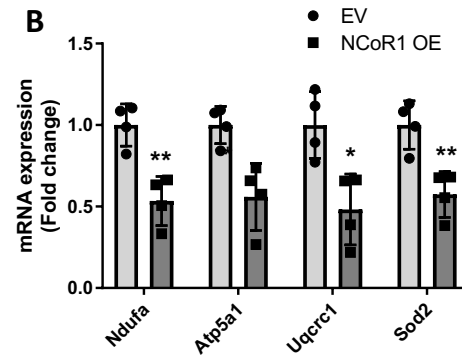

**C**

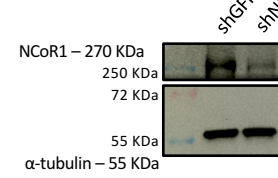

**D**

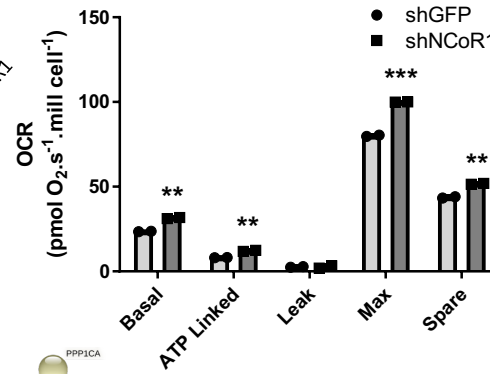

**E**

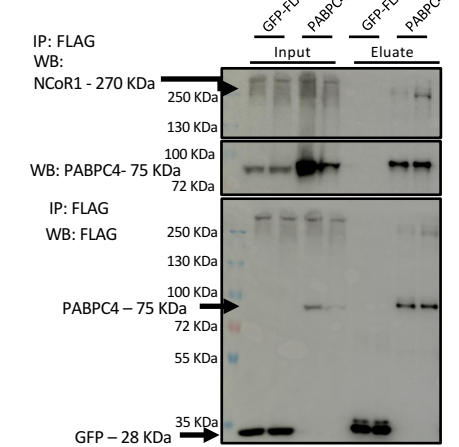

**F**

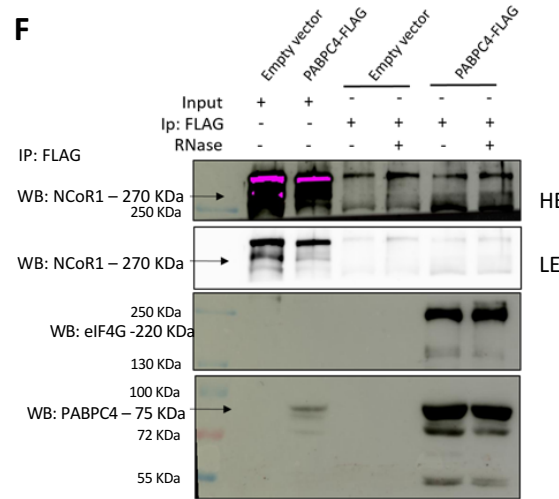

**G**

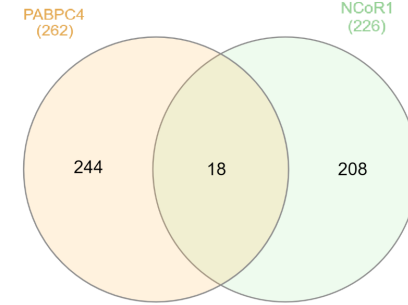

**H**

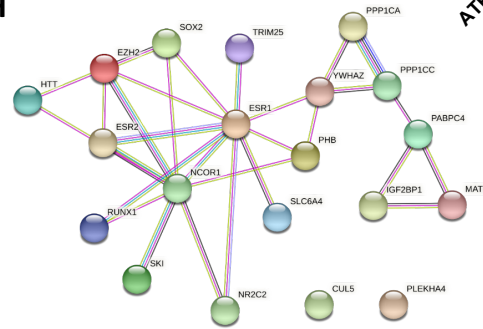

**I**

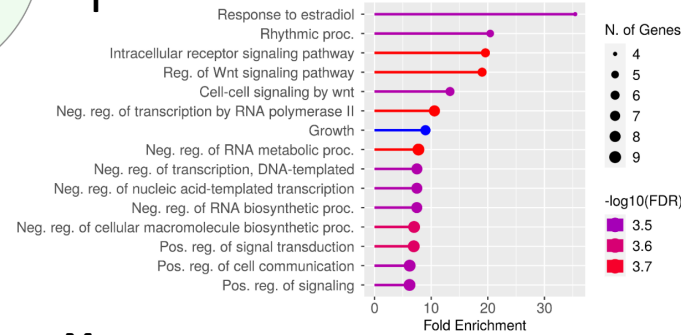

**J**

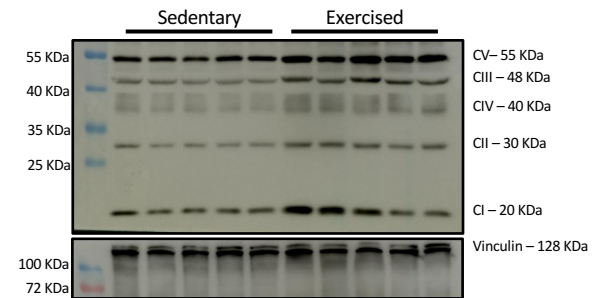

**K**

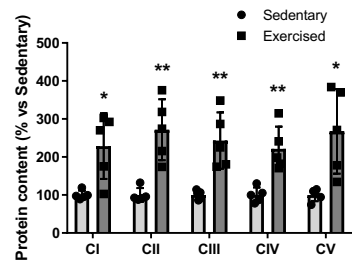

**L**

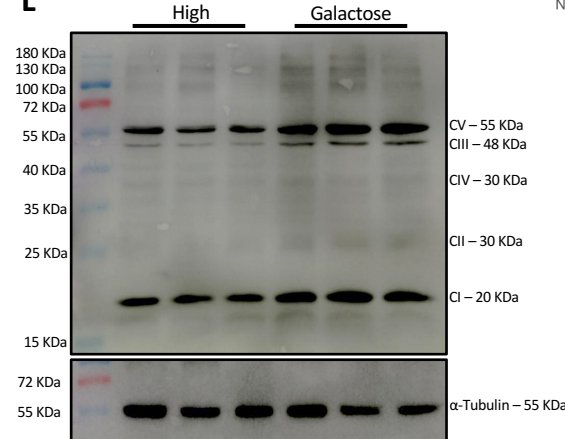

**M**

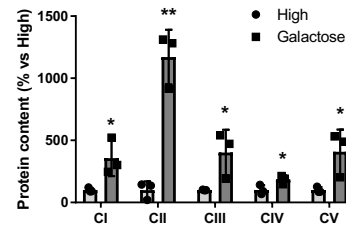

**N**

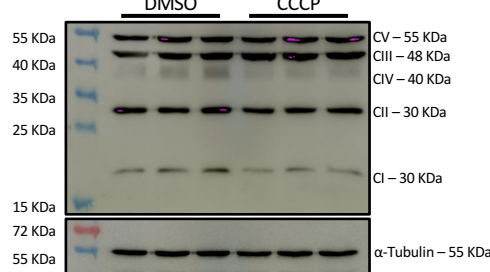

**O**

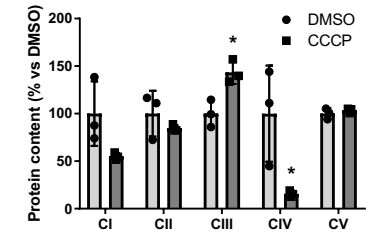

Supplement: Suppl Figure 1 [file mmc1.pdf]

Suppl. Figure 2

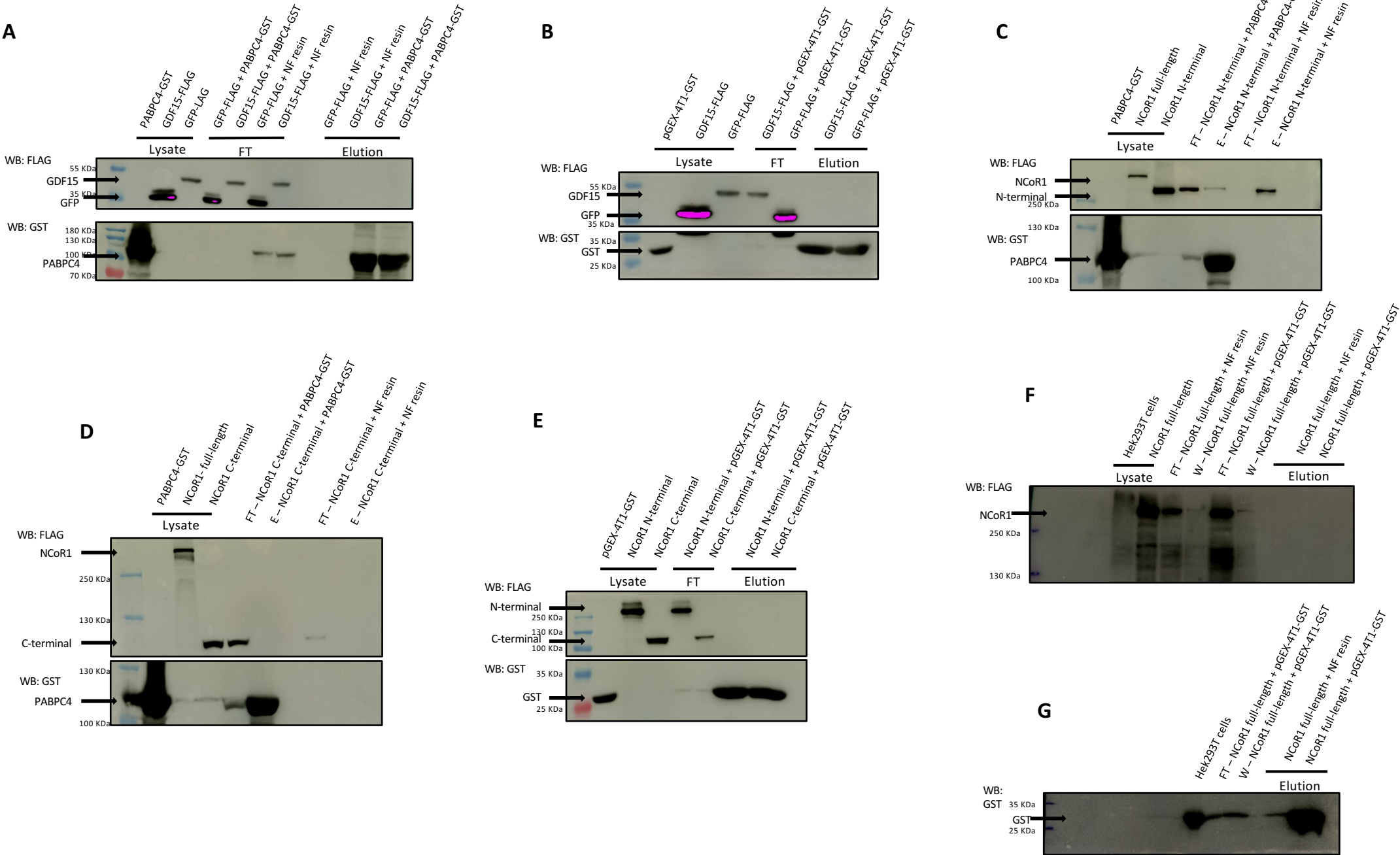

Supplement: Suppl Figure 2 [file mmc2.pdf]

Suppl. Figure 3

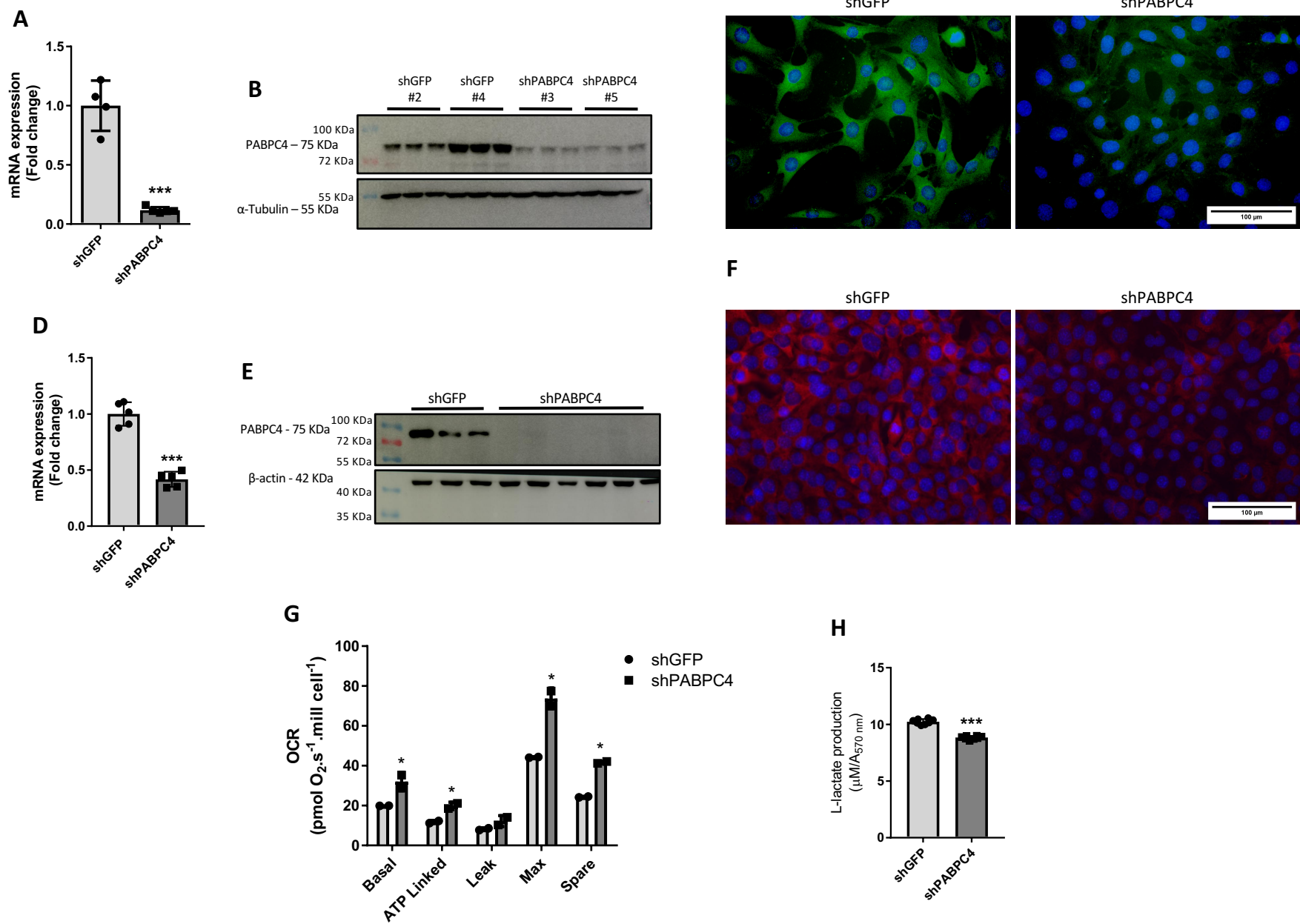

Supplement: Suppl Figure 3 [file mmc3.pdf]

Suppl. Figure 4

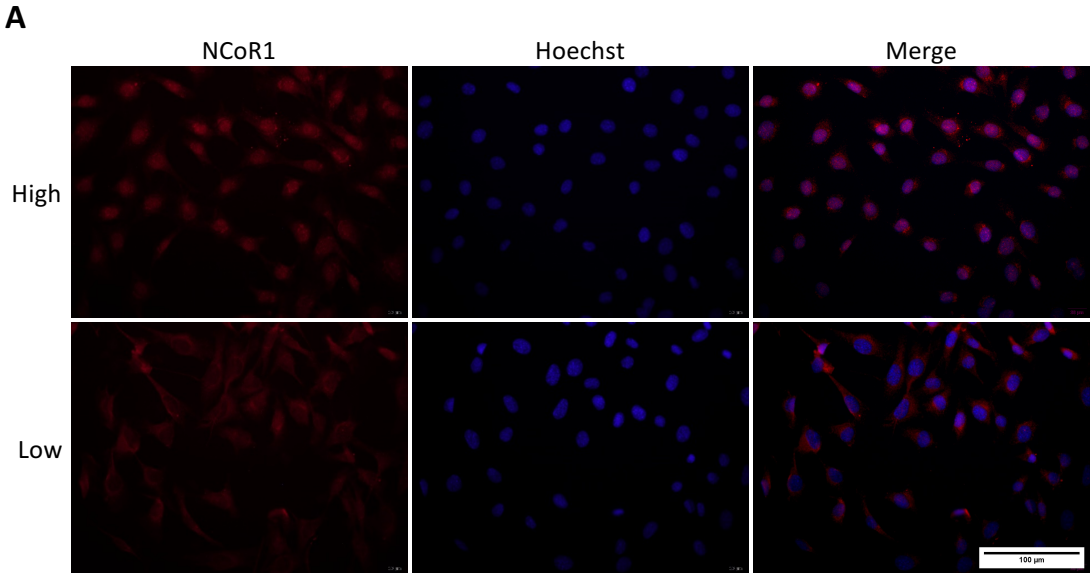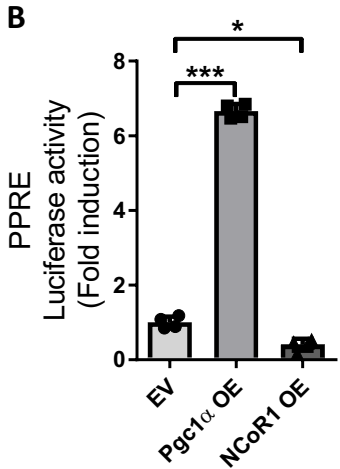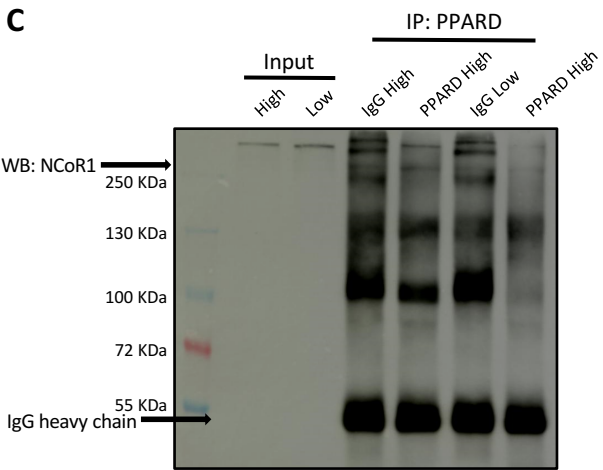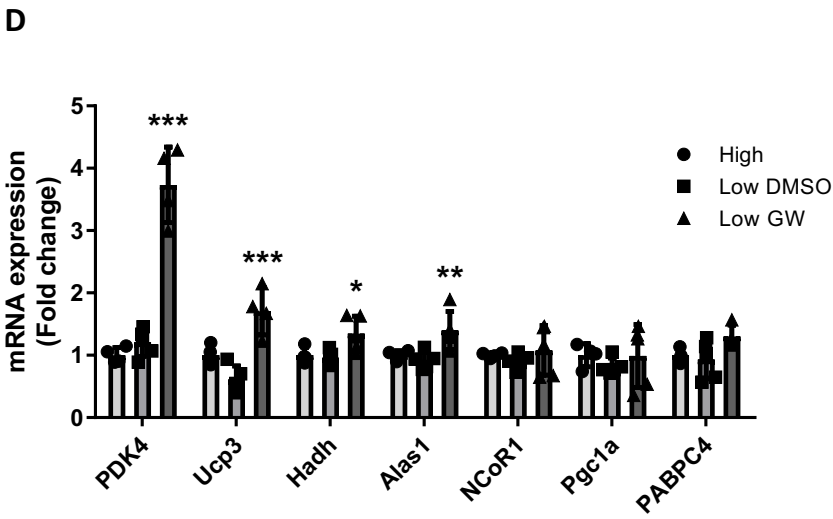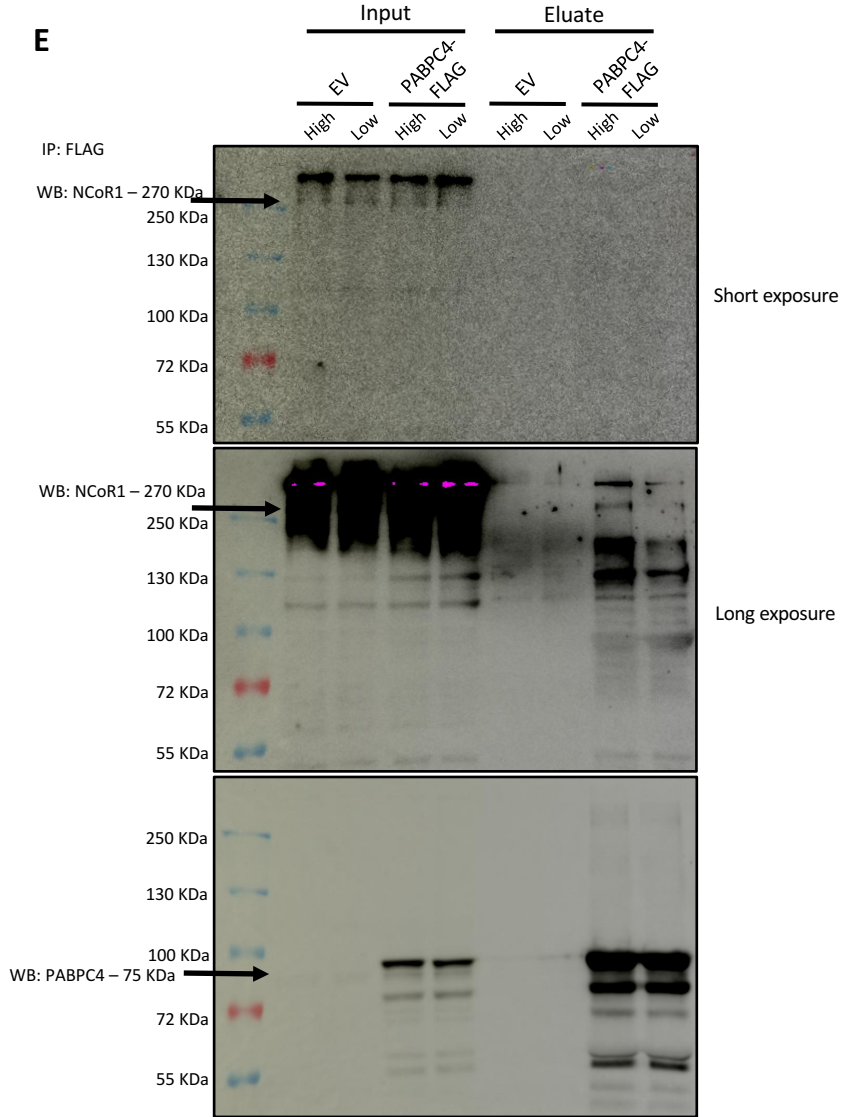

Supplement: Suppl Figure 4 [file mmc4.pdf]

Suppl. Figure 5

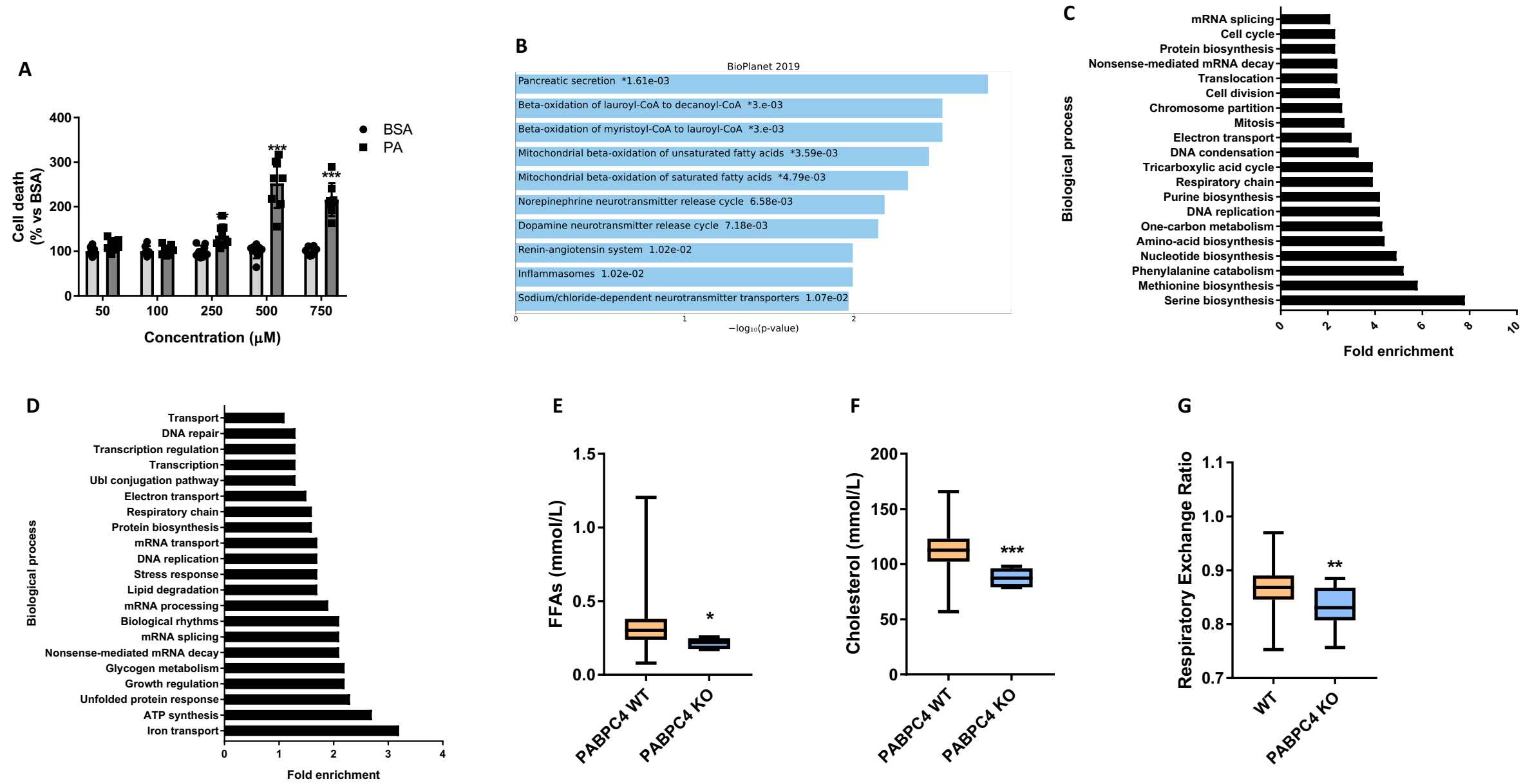

Supplement: Suppl Figure 5 [file mmc5.pdf]

**Suppl. Figure 5 continued**

H

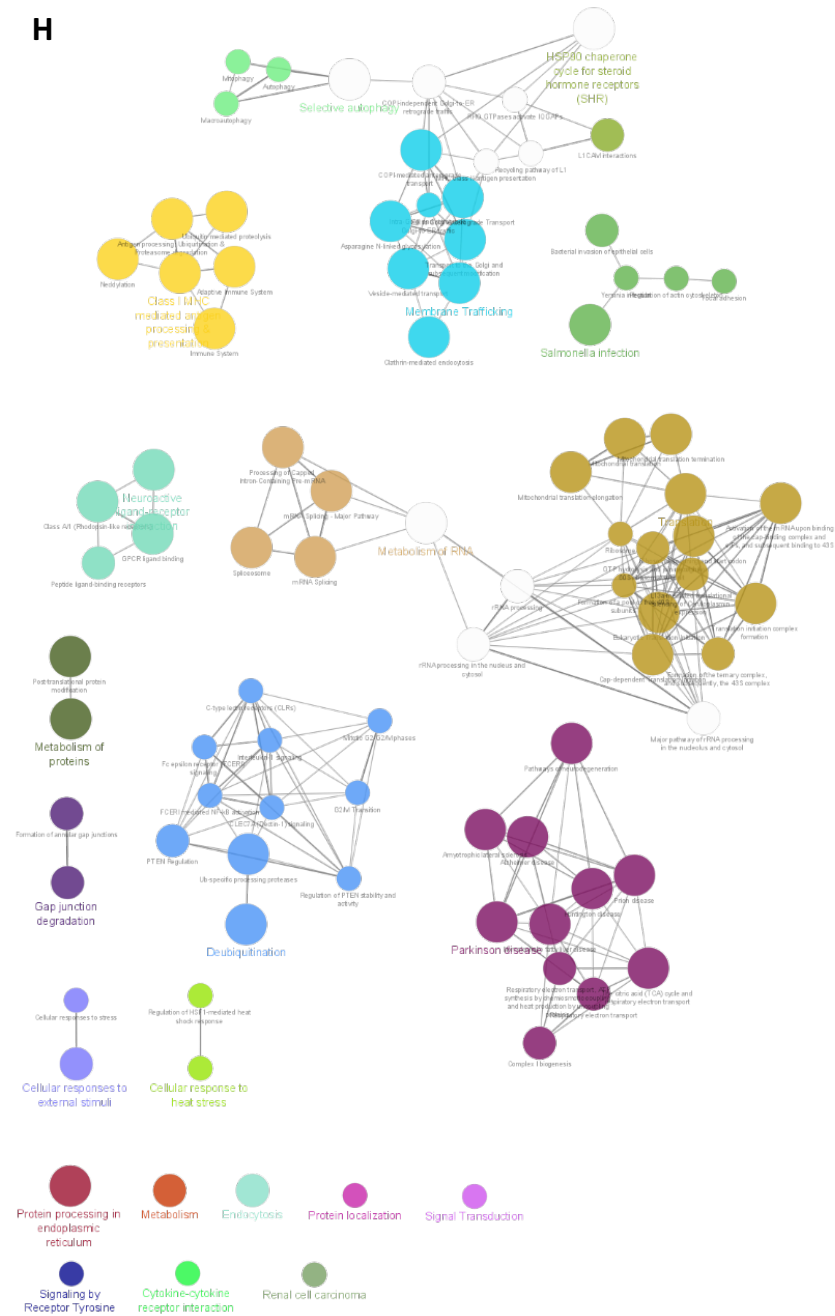

Supplement: Suppl Figure 5 CONTINUED [file mmc6.pdf]
